# The *AEROPILs* Generation: Novel Poly(Ionic Liquid)-Based Aerogels for CO_2_ Capture

**DOI:** 10.3390/ijms23010200

**Published:** 2021-12-24

**Authors:** Raquel V. Barrulas, Clara López-Iglesias, Marcileia Zanatta, Teresa Casimiro, Gonzalo Mármol, Manuela Ribeiro Carrott, Carlos A. García-González, Marta C. Corvo

**Affiliations:** 1 i3N|Cenimat, Department of Materials Science (DCM), NOVA School of Science and Technology, NOVA University Lisbon, 2829-516 Caparica, Portugal; r.barrulas@campus.fct.unl.pt (R.V.B.); zanatta@uji.es (M.Z.); 2 Department of Pharmacology, Pharmacy and Pharmaceutical Technology, I+D Farma Group (GI-1645), Faculty of Pharmacy and Health Research Institute of Santiago de Compostela (IDIS), Universidade de Santiago de Compostela, E-15782 Santiago de Compostela, Spain; clara.lopez.iglesias@rai.usc.es (C.L.-I.); carlos.garcia@usc.es (C.A.G.-G.); 3 LAQV-REQUIMTE, Chemistry Department, NOVA School of Science and Technology, NOVA University Lisbon, 2829-516 Caparica, Portugal; teresa.casimiro@fct.unl.pt; 4 LAQV-REQUIMTE, Instituto de Investigação e Formação Avançada, Departamento de Química e Bioquímica, Escola de Ciências e Tecnologia, Colégio Luís António Verney, Universidade de Évora, 7000-671 Evora, Portugal; gmd@uevora.pt (G.M.); manrc@uevora.pt (M.R.C.)

**Keywords:** polymeric ionic liquids, chitosan, aerogel, porosity induction, CO_2_ capture, adsorption

## Abstract

CO_2_ levels in the atmosphere are increasing exponentially. The current climate change effects motivate an urgent need for new and sustainable materials to capture CO_2_. Porous materials are particularly interesting for processes that take place near atmospheric pressure. However, materials design should not only consider the morphology, but also the chemical identity of the CO_2_ sorbent to enhance the affinity towards CO_2_. Poly(ionic liquid)s (PILs) can enhance CO_2_ sorption capacity, but tailoring the porosity is still a challenge. Aerogel’s properties grant production strategies that ensure a porosity control. In this work, we joined both worlds, PILs and aerogels, to produce a sustainable CO_2_ sorbent. PIL-chitosan aerogels (*AEROPILs*) in the form of beads were successfully obtained with high porosity (94.6–97.0%) and surface areas (270–744 m^2^/g). *AEROPILs* were applied for the first time as CO_2_ sorbents. The combination of PILs with chitosan aerogels generally increased the CO_2_ sorption capability of these materials, being the maximum CO_2_ capture capacity obtained (0.70 mmol g^−1^, at 25 °C and 1 bar) for the CHT:P[DADMA]Cl_30%_ *AEROPIL*.

## 1. Introduction

In light of the urgent climate change mitigation strategies, the development of a material capable of performing direct CO_2_ capture (CC) is highly desirable. CC commonly focuses on solvent scrubbing for CO_2_ chemical absorption. However, this process has several disadvantages which interfere with the energy efficiency and overall cost, like solvent losses through evaporation, the formation of corrosive by-products, and high energy consumptions during solvent regeneration. CC can also use solid physical adsorbers like zeolites, activated carbon, metal organic frameworks (MOFs), covalent organic frameworks (COFs), and organic/inorganic membranes, which have a diminished performance in the presence of impurities [1,2,3].

Ionic liquids (ILs), i.e., organic salts with melting points below 100 °C, result from the combination of organic cations with organic or inorganic anions, and have been proposed as alternative solvents for CC since they are stable, highly selective for CO_2,_ and recyclable. ILs can be adjusted towards CC due to the possibility of having multiple cation/anion combinations [3,4,5,6,7].

Poly(ionic liquid)s (PILs) join the unique characteristics of ILs and a macromolecular framework, providing enhanced CC in comparison to their monomer analogues [8]. One way to improve the sorption capacity is to modify their chemical structure (cation and anion), since it highly affects CO_2_ sorption capacities. Regarding the cations, higher performance was observed in ammonium derivatives, followed by pyridinium > phosphonium > imidazolium. Long alkyl substituents on cations and cross-linking can decrease CC performance of PILs due to steric hindrance. The sorption capacities of anions are in the order of acetate > tetrafluoroborate > hexafluorophosphate > bis(trifluoromethylsulfonyl)imide [3].

Other important features that should be considered are the porosity of the material and the specificity, which remain a challenge to solve. For this reason, it is relevant to develop PILs through a soft templating approach creating porous materials able to interact in a more specific manner with CO_2_ [9,10]. Aerogels, whose production can be considered as a particular case of soft templating, are a class of nanoporous materials generated through the removal of the pore fluid from gels without a significant damage in their 3D polymeric network structure. Aerogels have low density, high specific surface area, high porosity, a tailorable surface functionality, and good sorption capacity [10,11,12,13,14,15]. They are currently in the market and under research for multiple applications such as thermal insulation, metal-ion sorption, filtration and separation, oil-water separation, drug delivery, catalysis, and CC [12,16,17,18]. Recent research on aerogels for CC focused on tailoring their physicochemical properties, which is exemplified by amine modified SiO_2_ aerogels using (3-aminopropyl)triethoxysilane (APTES), polyethyleneimine (PEI), and tetraethylenepentamine (TEPA) [19,20,21,22]. Other alternatives are also being explored such as nitrogen-doped carbon aerogels, carbon nitride-functionalized porous reduced graphene oxide aerogels and glucose/graphene-based aerogels [23,24,25,26]. Moreover, the development of aerogels from polysaccharides is dramatically increasing since they are biocompatible, sustainable, renewable, and have a low toxicity. [10,14,27,28,29]. Namely, chitosan aerogels are relevant for CO_2_ sorption since the free amino groups of chitosan can covalently bond CO_2_ by a mechanism involving one CO_2_ molecule and two adjacent amine groups. It is important to highlight that these materials have potential for a selective gas sorption at low CO_2_ pressures, which is relevant for atmospheric pressure capture [12,27].

Some examples of bio-based sorbents and their respective CO_2_ capture capacities and specific surface areas are presented in Table 1—chitosan-based sorbents from entries 1–8 and cellulose-based sorbent from entries 9–13.

Recently, PIL-based aerogels were studied for applications such as protein enrichment and separation and solid-phase microextraction [11,42]. For example, PIL-based cellulose aerogels with 1-vinyl-3-aminopropyl imidazolium cations were applied for selective separation of a target protein from a real serum sample. In the end, the aerogels showed a well-interconnected porous structure with high porosity and high adsorption capacity towards bovine serum albumin [11].

**Figure 1:**
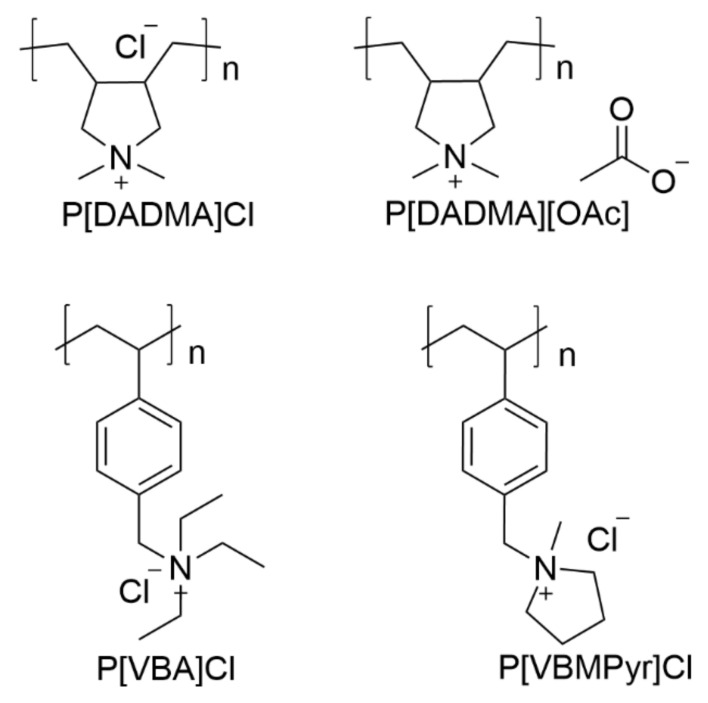
Chemical structures of the poly(ionic liquid)s used for *AEROPILs* formulations.

**Figure 2:**
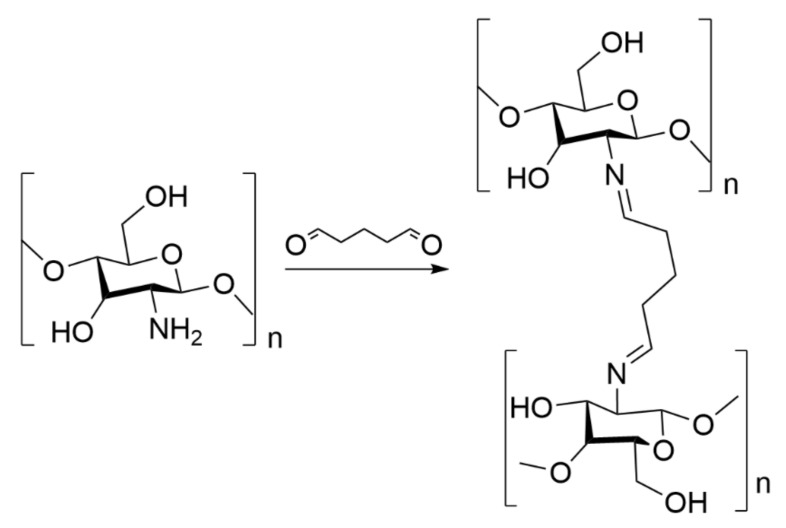
Cross-linking chitosan with glutaraldehyde.

To the best of our knowledge, PIL-based aerogels have not been developed so far for CC purposes; however, due to their characteristics, they are envisioned as promising materials for CO_2_ mitigation, especially in association with the well-known action of PILs towards CO_2_ [10]. Therefore, this work focused on the development of high-performance CO_2_ sorbents using a simple, easily scalable, cost-effective, and environmentally-friendly approach based on PIL-chitosan composite aerogel beads—*AEROPILs*. Aerogels were formulated with several PILs (Figure 1) and with glutaraldehyde as cross-linker. Chitosan is often cross-linked with glutaraldehyde (Figure 2) since this is one of the most efficient cross-linking agents [43,44]. After the proper morphological and textural characterization, their CO_2_ sorption capacities were evaluated.

## 2. Results and Discussion

### 2.1. Morphological and Textural Properties of the Chitosan Aerogels

The physicochemical properties of *AEROPILs* (Table 2 for selected samples; Table S1 for complete set of samples) as well as their textural properties (Table 3 for selected samples; Table S2 for complete set of samples) were studied. The dimensions of the beads were measured through the analysis of their digital images. The diameter and volume of the beads allowed the calculation of the volume shrinkage during each processing step. It is possible to observe that there was a volume shrinkage from the hydrogel to the aerogel varying between 58.1–74.7%, which was smaller for the aerogels with PIL and glutaraldehyde. Since the shrinkage can be attributed to the flexibility of the polymeric chains of the chitosan that are brought closer after solvent extraction [45], it can be inferred that the cross-linker is preventing this flexibility through the formation of imine bonds with the amine residues from chitosan, stabilizing the network structure. Additionally, a possible plasticizing effect of the PIL on the chitosan can be present due to the formation of hydrogen bonds [46]. The overall porosity was high, above 94.6% in all cases, with only slight differences between them. The skeletal densities of the chitosan/PIL aerogels measured by helium pycnometry were lower than the ρ_skel_ of the chitosan. This might be due to the skeletal density of PILs being lower than chitosan, which will decrease the total skeletal density, or because PILs induce smaller pores that are not accessible to the helium molecule [47]. However, in most cases, the cross-linked chitosan/PIL aerogels exhibited higher ρ_skel_ and pore size than the uncross-linked *AEROPILs*.

The textural properties (a_BET_, V_P,BJH_ and D_P,BJH_) of PIL-chitosan aerogel particles were obtained by nitrogen adsorption-desorption analysis (Table 3 for selected samples; Table S2 for complete set of samples). Overall, the specific surface areas and pore volume of the cross-linked chitosan/PIL aerogels—CHT:Glut_0.30%_:P[DADMA][OAc]_15%_ and CHT:Glut_0.30%_:P[DADMA]Cl_15%_ (Table 3, entries 11,13 respectively)—increased in comparison to pure chitosan aerogels (CHT), which is interesting for the final application. For PILs functionalized with the vinylbenzyl moiety, a slight decrease of the specific surface area is observed, in comparison with P[DADMA]Cl and P[DADMA][OAc]. The structural difference between P[DADMA] and the PILs with vinylbenzyl moiety, suggests that each might establish different interactions with the chitosan matrix and solvents (H_2_O/ethanol/CO_2_) during the aerogel formulation. Given that P[DADMA] PILs are much more hydrophilic, they can have a higher affinity towards chitosan, and this may be the reason for the possibility of achieving a higher porosity induction in the final material. Additionally, the introduction of the cross-linker enhances the specific surface area. The contribution of the mesopore (2–50 nm) and macropore (>50 nm) volumes to the total pore volume was studied (V_mes_ and V_MP_ in Table 3, respectively). Results indicate that the V_MP_ is predominant (above 84% in all cases). Moreover, all aerogels exhibit isotherms with a composite behaviour between type IV and type II, according to IUPAC definitions (Figures S5–S25), with hysteresis indicating mesoporous materials, suggesting that the pore network has macropores not entirely filled by pore condensate [48]. According to the BJH method, the mesoporous size distribution has a unimodal distribution [45]. This dual porosity of PIL-chitosan aerogels can be of relevance for CO_2_ capture and conversion, since macropores and mesopores enhance diffusion and accessibility of active sites by guest molecules [10].

**Figure 3:**
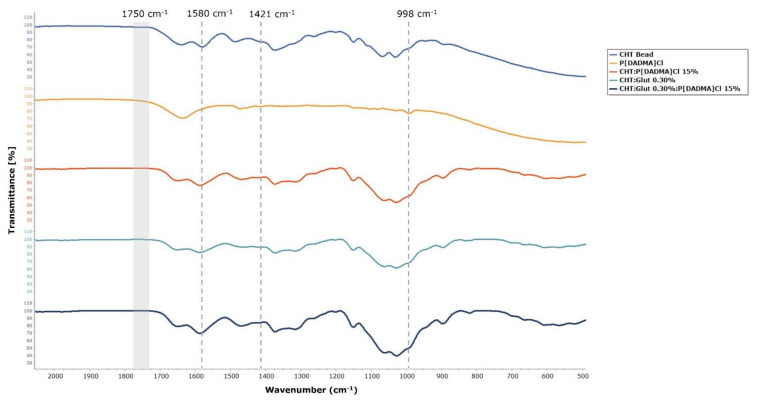
ATR–IR spectra of (blue) CHT bead, (orange) P[DADMA]Cl, (red) CHT:P[DADMA]Cl_15%_ bead, (cyan) CHT:Glut_0.30%_ bead, and (dark blue) CHT:Glut_0.30%_:P[DADMA]Cl_15%_ bead.

ATR–FTIR spectra (Figure 3) confirms the PIL incorporation into the aerogel and that the cross-linking of chitosan with glutaraldehyde was successfully achieved. The vibration band at 1750 cm^−1^ corresponding to the C=O carbonyl group of free glutaraldehyde is absent. Any unreacted glutaraldehyde is expected to be removed in the solvent exchange with ethanol followed by scCO_2_ drying, because this compound is soluble in supercritical mixtures of ethanol/CO_2_. Instead, an increased vibration at 1580 cm^−1^ appears corresponding to the imine C=N bond formed between the amine residues of CHT and the aldehyde terminals of the glutaraldehyde, adding to the amide II vibration from CHT [43]. Additionally, it is possible to notice some changes in the region around 998 cm^−1^ with the addition of PIL, due to the CH vibration of the PIL’s backbone. Around 1421 cm^−1^,there is also a characteristic vibration of PIL corresponding to -CH in–plane bending [49].

**Figure 4:**
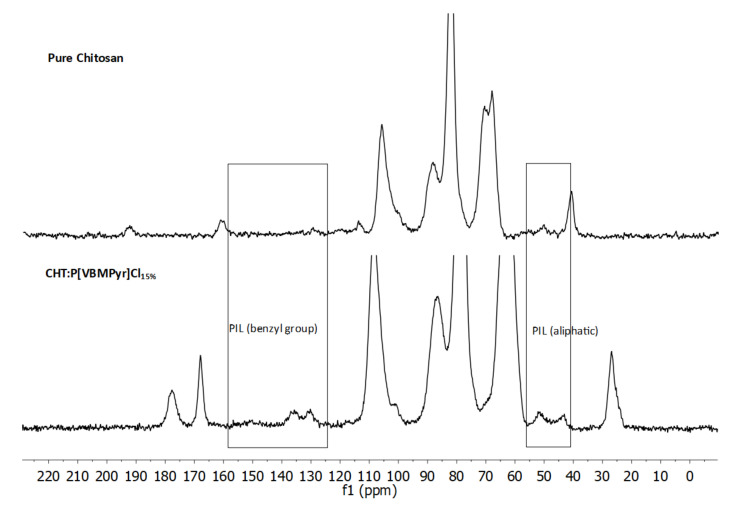
^13^C CP-TOSS NMR spectra of pure chitosan and CHT:P[VBMPyr]Cl_15%_ beads, respectively.

^13^C CP-TOSS NMR spectra (Figure 4 and Figure S4) also confirms the PIL incorporation into the aerogel and the cross-linking of chitosan with glutaraldehyde. These differences are observable in the aromatic region (125–155 ppm) due to the benzyl substituent and the aliphatic region (40–55 ppm) due to the amine alkyl substituents.

**Figure 5:**
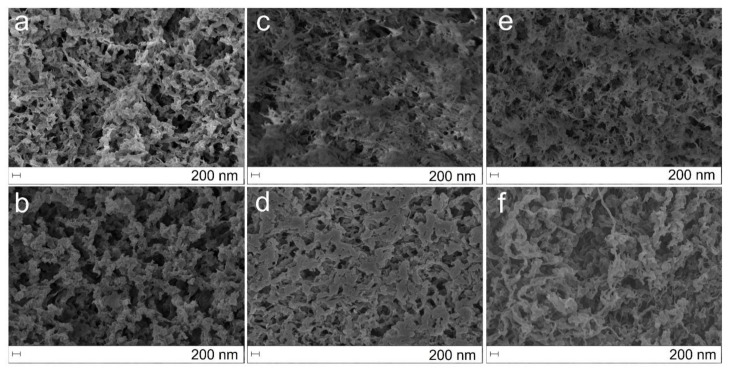
Textural appearance of the (**a**,**c**,**e**) interior of beads CHT:Glut_0.30%_, CHT:Glut_0.30%_:P[DADMA][OAc]_15%_, CHT: P[DADMA][OAc]_15%_, respectively, and (**b**,**d**,**f**) surface of beads CHT:Glut_0.30%_, CHT:Glut_0.30%_:P[DADMA][OAc]_15%_, CHT: P[DADMA][OAc]_15%_, respectively, by SEM imaging (scale bar: 200 nm).

The most promising beads which presented higher specific surface areas were analysed through SEM imaging. In Figure 5, images of *AEROPILs* containing P[DADMA][OAc] with and without cross-linker (glutaraldehyde) are presented. Images of chitosan beads with the same amount of cross-linker are also provided for comparison (Figure 5a,b). The dual porous structure was confirmed in the inner and outer structure of the particles. It is possible to observe that the presence of the cross-linker leads to more structured fibers, with an associated decrease in pore diameter. The presence of the PIL also changes the fibers since it appears to coat them (Figure 5c–f). Furthermore, comparing with the results described by López-Iglesias et al. [45], it is possible to confirm that both the presence of PIL and PIL/cross-linker induced changes in the fibers structure and organization (also confirmed by the SEM images of the other conditions—Figures S26–S42).

### 2.2. CO_2_ Capture

CO_2_ adsorption-desorption was evaluated for the aerogels after being heated under He at 120 °C, as the representative TGA curves presented in Figure S43 show that decomposition of the materials did not occur at this temperature. The adsorption-desorption results of chitosan aerogel beads and *AEROPILs* at 25 °C and a pressure of 1 bar (Figures S44 and S45) indicate that the materials were able to adsorb as well as retain CO_2_. In fact, it can be seen that when the gas was changed from He to CO_2_, adsorption was initially fast, but then the adsorbed amount increased gradually; that is, after exposure to 100 mL min^−1^ flow of CO_2_ for 10 min, a plateau was not reached. This suggests that higher adsorbed amounts would be obtained with a longer exposure time.

Analysing the CC capacity obtained with chitosan beads (0.57 mmol g^−1^) with the CC reported for pure chitosan (0.02 mmol g^−1^) [30], it is possible to state that by achieving a 3D morphology, there is a pronounced effect on CC capacity. This is most likely a consequence of the increase in the specific surface area and porosity, resulting in a CC even higher that the previously obtained by Alhwaige et al. [32] with other chitosan aerogels, which was 0.40 mmol g^−1^. Our chitosan aerogel achieved an increment on CO_2_ adsorption of approximately 29 times, compared to pure chitosan. This is the main reason for the increasing interest on porous materials research, especially in CC [10].

*AEROPILs* had a good CO_2_ capture capacity in comparison with data in the literature regarding porous PILs, since for example the porous PILs synthesized by Sun et al. [50] presented a poor CO_2_ capture capacity after the same 10 min of exposure to CO_2_ (approximately 15 mg g^−1^—original value—or 0.34 mmol g^−1^—calculated value). Moreover, when the gas is changed back to He, part of the CO_2_ desorbs quickly but most of it is retained after a long period under 100 mL min^−1^ flow of He. The overall behaviour is possibly due, at least partially, to carbamate formation. This possibility of carbamate formation upon contact with CO_2_ is supported by the presence of a chemical shift around 165 ppm in the ^13^C CP-TOSS NMR spectrum (Figure 4—CHT:P[VBMPyr]Cl_15%_) which indicates carbamate formation during supercritical CO_2_ drying of the aerogels. After heating to 120 °C, the second sorption cycles had a profile broadly similar to that of the first, but the adsorbed amounts were about 82–97% of those obtained in the first cycle, indicating that some changes in the materials occurred. Nevertheless, the materials still adsorbed good amounts of CO_2_ and were also able to retain most of it for a long period.

The attempts to relate the CC performance of *AEROPILs* with the previously determined morphological properties (Table 2) revealed a complex relationship. The overall porosity obtained in these materials was highly dependent on the aerogel formulation, and not on the PILs identity; the skeletal density (1.248–1.421 g/cm^3^), envelope density (0.046–0.072 g/cm^3^), and overall porosity (94.6–96.4%) exhibited a range of values characteristics of aerogels, with a noticeable effect of PILs with vinylbenzyl moiety that displayed, in most of the cases, a lower superficial area and overall porosity. These properties are reflected in a non-linear manner in the CC capacity. As expected, the lower the envelope density, the higher the overall porosity, which achieved a higher CC capacity, as is the case for CHT:Glut_0.30%_, CHT:Glut_0.30%_:P[DADMA][OAc]_30%,_ CHT:Glut_0.30%_:P[DADMA]Cl_15%_ (Table 4, entries 10, 12, 13, respectively). However, it’s possible to also find a higher envelope and skeletal density, a lower overall porosity, but a better CC performance, as is the case of CHT:P[DADMA]Cl_30%_ (Table 4, entry 6).

Among the several *AEROPILs*, the largest CO_2_ adsorbed amount obtained after exposure to CO_2_ for 10 min was 0.70 mmol g^−1^ (CHT:P[DADMA]Cl_30%_—Table 4, entry 6), which corresponds to 35-fold higher than pure chitosan (0.02 mmol g^−1^) [30]. Comparing with other porous PILs, for example the ones described by Eftaiha et al. (0.59 mmol g^−1^ at 25 °C and 1 bar) [51], our material poses a promising CO_2_ sorption capacity. Additionally, the amount of PIL (maximum of 30% *w*/*w* with respect to chitosan) used on *AEROPILs* was lower than for the materials described by Esko et al. (IL or ionogel content between 40–80 wt%) [36] and Ding et al. (80 wt% COF-IL) [35]. Lowering the amount of PIL needed in these formulations represents an economical advantage. Moreover, *AEROPILs* formulation is very straightforward. After PIL synthesis, aerogels are obtained using a simple sol-gel process, given that the PIL properly stabilized into the aerogel.

The best CO_2_ adsorption result was obtained for the *AEROPIL* beads prepared with the commercial PIL P[DADMA]Cl (CHT:P[DADMA]Cl_30%_—Table 4, entry 6), not requiring additional synthesis steps. Contrary to the expected, *AEROPILs* with P[DADMA]Cl presented higher CO_2_ adsorption capacities comparing with P[DADMA][OAc]. This is an indication that not only is the chemical entity relevant for the performance of the final material, but a balance between the chemical functionalities and the morphological parameters must exist [10].

The CO_2_ capture capacities obtained with *AEROPILs* are comparable with the SILP ionogels (Table 1, entry 7). However, this comparison is not straightforward, as the same pressure and temperature conditions are not always available for all the materials. In the present work the amount of PIL in *AEROPILs* was much lower than the amount of IL employed in SILPs, for a comparable capture efficiency.

**Figure 6:**
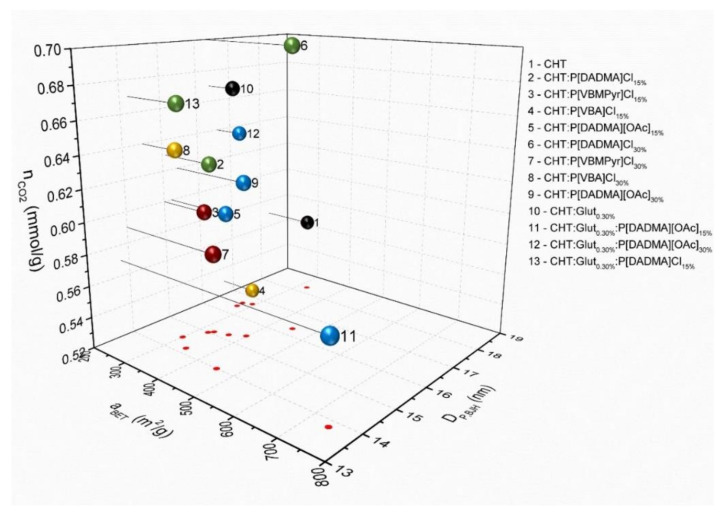
Correlation between CO_2_ capture capacities of *AEROPILs*, specific surface area and mean pore diameter. Notation: n_CO2_, CO_2_ capture capacity; D_P,BJH_, mean pore diameter; a_BET_, specific surface area. Chitosan aerogels without PIL (black); P[DADMA]Cl *AEROPILs* (green); P[VBMPyr]Cl *AEROPILs* (red); P[VBA]Cl *AEROPILs* (yellow); P[DADMA][OAc] *AEROPILs* (blue).

Analysing Figure 6, a higher PIL content resulted in higher CO_2_ sorption with a more noticeable effect for PIL chlorides than for PIL acetates. *AEROPILs* cross-linking generally increased CO_2_ sorption, except for P[DADMA][OAc]_15%_. Although in the absence of cross-linker P[DADMA][OAc] *AEROPILs* exhibited only a slight increase in CO_2_ sorption when compared to the chitosan aerogel (CHT:P[DADMA][OAc]_15%_ and CHT:P[DADMA][OAc]_30%_—Table 4, entries 5, 9 respectively), in the presence of cross-linker, the amount of CO_2_ uptake was enhanced for the higher amount of PIL (CHT:Glut_0.30%_:P[DADMA][OAc]_15%_ and CHT:Glut_0.30%_:P[DADMA][OAc]_30%_—Table 4, entries 11, 12 respectively).

Overall, the next generation of *AEROPILs* should target a higher specific surface area not necessarily at the expense of increasing PIL concentration. However, a higher amount of PIL stabilized inside the bead should make a difference, because from the CO_2_ sorption results obtained, we conclude that sorption is taking place mostly in the interior of the beads, possibly through increased diffusion.

In addition to the CO_2_ capture capacity, there is a good indicator that the *AEROPIL* beads can be promising for CO_2_ catalysis due to the possibility of reacting with CO_2_ [52]. The use of CO_2_ as a C1 building block to produce other chemical products is considered a clean and efficient approach for the reuse of this gas, since it can minimize the need for consumption of sensitive and toxic carbon sources [53]. Despite the efforts dedicated to this transformation, only small improvements on the catalytic activity have been achieved so far. Additionally, the direct capture and conversion using the same material that works as sorbent and catalyst is still under research.

## 3. Materials and Methods

### 3.1. Materials

Chitosan (deacetylation degree 75–85%, viscosity 20–300 mPa·s, Mw 50–190 kDa), glutaraldehyde solution (grade II, 25 wt.% in H_2_O), 4-vinylbenzyl chloride (90% purity), poly(diallyldimethylammonium chloride) (P[DADMA]Cl) solution (20 wt. % in H_2_O, viscosity 250–500 mPa·s, average Mw 200,000–350,000 medium molecular weight), 1-methylpyrrolidine (≥ 98.0% purity), triethylamine (≥ 99.5% purity), and anion exchange resin Amberlyst A-26 (OH^-^ form) were supplied by Sigma-Aldrich (St. Louis, MO, USA). 2,2′-Azobis(2-methylpropionitrile) (AIBN) was purchased from Glentham Life Sciences (Corsham, UK). Glacial acetic acid and absolute ethanol (EtOH) were both purchased from VWR (Radnor, PA, USA). NaOH (98% purity) was purchased from Panreac (Barcelona, Spain). DMSO-*d_6_* was purchased from Euriso-top (Saint-Aubin, France). Water was purified using reverse osmosis (resistivity > 18 MΩ.cm, Milli-Q, Millipore^®^, Madrid, Spain). Carbon dioxide (99.8% purity) was supplied by Nippon Gases (Madrid, Spain). He 4.6 and CO_2_ 4.5, supplied by Linde Portugal, were used in thermogravimetric analysis (TGA) and CO_2_ capture experiments. All chemicals were used without further purification.

### 3.2. IL and PIL Synthesis

ILs: *p*-vinylbenzyltriethylammonium chloride ([VBA]Cl) and 1-methyl-1-(4′-vinylbenzyl)pyrrolidinium chloride ([VBMPyr]Cl) were synthesized according to the experimental details provided in Supporting Information (Section 1) [54,55].

PILs: poly(*p*-vinylbenzyltriethylammonium) chloride (P[VBA]Cl) and poly(1-methyl-1-(4′-vinylbenzyl)pyrrolidinium) chloride (P[VBMPyr]Cl) were synthesized according to the experimental details provided in Supporting Information (Section 2) [56]. Poly(diallyldimethylammonium) acetate (P[DADMA][OAc]) was obtained through anion exchange reaction of the corresponding chloride salt as described in Supporting Information (Section 2.1) [57,58].

### 3.3. Preparation of Chitosan Aerogel Beads

#### 3.3.1. Preparation of Chitosan Hydrogel

Hydrogel particles were prepared according to a sol-gel method, based on the procedure described by López-Iglesias et al. [45]. Firstly, chitosan (2.5% *w*/*v*) was dissolved in Milli-Q water with 1% (*v*/*v*) of acetic acid to obtain 30 mL of solution. The solution was mechanically stirred for 8 h, and then left to settle until the disappearance of the gas bubbles formed during the stirring. Solutions of PIL (15% and 30% *w*/*w* with respect to chitosan) and glutaraldehyde (0.13% to 4.00% *w*/*w* with respect to chitosan) were added at this point after the chitosan was completely dissolved. PILs were previously dissolved in 3 mL of water, volume that was subtracted to the total water volume used in the chitosan solution preparation. Glutaraldehyde (100–1500 µL), when used, was added directly to the chitosan solution. Subsequently, batches of 15–18 mL of the resulting chitosan solution were transferred to a plastic syringe (nozzle diameter of 2 mm) and added dropwise to 100 mL of a NaOH 1 mol L^−1^ gelation bath, using a syringe pump (AL-1000, World Precision Instruments, Sarasota, FL, USA) at a constant flow rate of 0.65 mL min^−1^. The distance from the syringe to the surface of the gelation bath was ca. 16.5 cm. Droplets gelified just after contact with the NaOH 1 mol L^−1^ solution, and hydrogel beads were formed. These beads were left in the gelation bath for 24 h. For the sake of comparison, beads without PIL and glutaraldehyde were produced in the same way.

#### 3.3.2. Solvent Exchange

The gelation bath was poured out of the beaker with the gel beads and immediately replaced by 100 mL of absolute EtOH. After 4 h, a second solvent exchange with a similar volume of EtOH was made, to remove any trace of water from the gel particles.

#### 3.3.3. Supercritical Extraction of the Gel Solvent

Alcogel particles were placed into Whatman paper cartridges and transferred to a 400 mL autoclave of the supercritical drying equipment (Thar Process, Pittsburgh, PA, USA). 100 mL of EtOH were previously added to the pressurized vessel to avoid early evaporation of the EtOH contained in the alcogels before reaching the supercritical conditions of the CO_2_-EtOH mixture [45,59]. A scCO_2_ flow of 5 g min^−1^ passed through the autoclave where the gels were contained at a processing temperature of 40 °C and pressure of 120 bar for 3.5 h. The extracted liquid ethanol was sampled at selected drying times to monitor the kinetics of the supercritical process (Figure S3).

**Figure 7:**
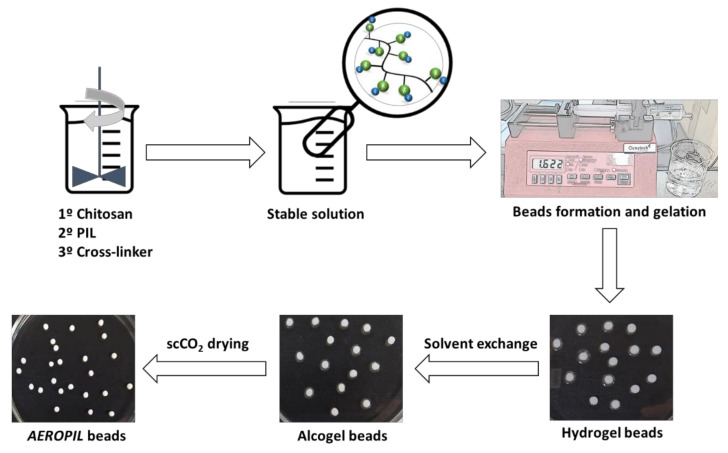
General scheme of *AEROPIL* beads preparation procedure.

Samples are addressed as in the general symbology: CHT:Glut_y%_:PIL_x%_ (where *x* is the percentage of PIL added and *y* is the percentage of glutaraldehyde added, both with respect to chitosan). The general scheme of *AEROPIL* beads preparation procedure is stated on Figure 7.

### 3.4. Morphology and Textural Properties

Images of hydrogel, alcogel, and aerogel chitosan beads were taken with a digital camera and analysed with ImageJ v1.53e software (U.S. National Institutes of Health, Bethesda, MD, USA) to measure the diameter and volume of the beads and thus to calculate the volume shrinkage during each processing step. Values were obtained from the analysis of minimum ca. 12 beads. The envelope density (ρ_env_) of the aerogel beads was calculated as the ratio between the average particle weight obtained with a precision balance (80A-200 M, Precisa, Dietikon, Switzerland) and the dimensions obtained by image analysis.

Skeletal density (ρ_skel_) was measured by helium pycnometry (MPY-2, Quantachrome, Delray Beach, FL, USA) at 25 °C and 1.03 bar from five replicates. The overall porosity (ε) of the dried gels was expressed in percentage and calculated according to Equation (1):

(1) $$\varepsilon =\left(1-\frac{\rho_{env}}{\rho_{skel}}\right)\times 100$$

The surface structure of the aerogel beads was also studied by SEM at 3 kV with a secondary electron detector (SEM, EVO LS15, Zeiss, Oberkochen, Germany). Aerogels were previously sputter coated (Q150 T S/E/ES, Quorum Technologies, Lewes, UK) with a thin layer (10 nm) of iridium to improve the contrast.

The textural properties of the aerogel beads were characterized by nitrogen adsorption-desorption analysis (ASAP 2000, Micromeritics, Norcross, GA, USA). The Brunauer−Emmet−Teller (BET) and the Barrett−Joyner−Halenda (BJH) methods were applied to calculate the specific surface area (a_BET_) and the pore size distribution, respectively [60]. The overall specific pore volume (V_p,BJH_) and the mean pore diameter (D_p,BJH_) were also obtained from the BJH method. The specific mesopore volume (V_mes_) was obtained from the cumulative BJH pore volume profiles of the aerogels in the mesopore range (2–50 nm). The specific volume occupied by the macropores (V_MP_) in the aerogels (range > 50 nm) was calculated as the difference between the total specific pore volumes of the aerogels (i.e., the inverse of the envelope density) and the specific pore volume occupied by mesopores (V_mes_).

For the chitosan and PIL-loaded chitosan aerogel beads, ATR-FTIR spectra were acquired using a Gladi-ATR accessory equipped with a diamond crystal (Pike, Madison, WI, USA) with 32 scans per spectrum with a spectral resolution of 2 cm^−1^, covering the 4000 to 400 cm^−1^ range. The samples were placed in the sample holder directly in the IR laser beam. Spectra were processed using SpectraGryph 1.2.15 software.

### 3.5. Solid-State NMR Spectroscopy

Solid state ^13^C MAS NMR spectra were acquired in a 11.7 T (500 MHz) AVANCE III Bruker spectrometer operating at 125 MHz (^13^C), equipped with a BBO probe head. The samples were spun at the magic angle at a frequency of 5 kHz, using 4 mm diameter rotors at room temperature. The ^13^C MAS NMR experiments were acquired with proton cross polarization and total suppression of sidebands (CP-TOSS) with a contact time of 2.0 ms, and a recycle delay of 5.0 s.

### 3.6. Thermogravimetric Analysis and CO_2_ Capture Experiments

TGA and CO_2_ capture experiments were performed using a PerkinElmer STA6000 thermogravimetric analyser controlled by PYRIS v.9.1 software. TGA curves were obtained with a heating rate of 10 °C min^−1^ and under a 20 mL min^−1^ flow of He. Before the CO_2_ capture tests, the samples were heated at 120 °C under a 100 mL min^−1^ flow of He. After cooling to 25 °C under He, the experimental procedure for the CO_2_ capture was the following: the sample was under a 100 mL min^−1^ flow of He for 10 min; then, the gas was switched to pure CO_2_ (100 mL min^−1^) and left for 10 min; afterwards, the gas was switched back to He (100 mL min^−1^) and left for 40 min. Subsequently, the sample was heated at 120 °C under He, cooled to 25 °C and a second cycle of adsorption-desorption was performed. Blank runs with no sample were carried out in order to correct for the change in buoyancy between He and CO_2_.

## 4. Conclusions

PIL-chitosan aerogels were successfully obtained with high porosity and surface areas. The PILs were stabilized into the aerogel in a simple and straightforward procedure, representing an advance in the formulation methodology, which is important to avoid PIL leakage. The introduction of the cross-linker enhanced the specific surface area, and led to more structured fibers, with an associated decrease in pore diameter.

The study of the CO_2_ capture ability of the chitosan aerogels highlighted the efficiency of the morphology. Aerogels from chitosan show an increased CO_2_ sorption compared to pure chitosan. The introduction of PILs in chitosan aerogels generally increases the CO_2_ sorption capability of these materials, being the maximum CO_2_ capture capacity obtained for the CHT:P[DADMA]Cl_30%_ *AEROPIL* (0.70 mmol g^−1^, at 25 °C and 1 bar). In general, a higher PIL content resulted in higher CO_2_ sorption, given that this effect is more pronounced for PIL chlorides than for PIL acetates. Additionally, an increased CO_2_ sorption could be observed for cross-linked *AEROPILs*.

Overall, porous PILs have been accomplished and the pioneering processing of *AEROPILs* has been herein established. There is still a challenge regarding the enhancement of specific surface areas with these materials. However, they are promising in the path toward a sustainable world. Further studies are being pursued to obtain PIL-chitosan aerogel beads with different PIL moieties to evaluate the cation and anion influence on CO_2_ capture and conversion.

## Figures and Tables

**Table 1:** Comparison of the CO_2_ capture capacities and respective specific surface areas reported for different bio-based sorbents.

Entry	Material	Modifications	n_CO2_ (mmol g^−1^)	S_BET_ (m^2^/g)	*P* (bar)	T (°C)	Refs.
1	Pure chitosan	-	0.02	nd	nd	nd	[30]
2	CHT-GO aerogels	CHT grafted GO	0.26	33.32	1.00	25	[31]
3	CHT-GO-20%	CHT-GO aerogels	4.15	412.00	1.00	25	[32]
4	QCHT/PVA aerogels	Quaternized CHT+ PVA	0.18	nd	nd	20	[33]
5	CHT-TPPS	Ionic complexation	0.90	26.75	5.00	25	[34]
6	COF-IL@CHT aerogel	COF-CHT aerogel + allylimidazolium IL	1.05 *	103.30	1.00	25	[35]
7	40%([EMIM][OAc] + 5%CHT) +60% silica	SILP—encapsulation of ionogel with nanoporous fumed silica	0.71	53.00	1.00	40	[36]
8	40%([BMIM]Cl + 5%CHT) +60% silica		0.11	52.00	1.00	40	
9	CNF + APS	Cellulose nanofibril aerogel grafted with aminosilane	1.91	51.80	1.00	25	[37]
10	CNC + APS	CNC aerogel grafted APS	1.50	29.14	1.00	25	[38]
11	CNF-X-a-CNC	Acetylated cellulose nanocrystals aerogels	1.14	21.04	1.00	0	[39]
12	APMDS-CNF	APMDS-modified CNF aerogel	1.01	nd	0.15	25	[40]
13	PCC-1	PEI-cross-linked cellulose aerogel	2.31	234.20	nd	25	[41]

Table abbreviations: nd, no data; n_CO2_, CO_2_ capture capacity; S_BET_, specific surface area; CHT, chitosan; GO, graphene oxide; PVA, poly(vinyl alcohol); TPPS, meso-tetrakis(4-sulfonatophenyl)porphyrin; COF, covalent organic framework; [EMIM][OAc], 1-ethyl-3-methylimidazolium acetate; [BMIM]Cl, 1-butyl-3-methylimidazolium chloride; SILP, inverse supported ionic liquid phase; CNF, cellulose nanofibrils; APS, 3-(2-aminoethylamino)-propylmethyldimethoxysilane; CNC, cellulose nanocrystal; APMDS, *N*-(2-aminoethyl)-3-aminopropylmethyldimethoxysilane; PEI, polyethylenimine; PCC, PEI-cross-linked cellulose. * Original data: 25.83 cm^3^ g^−1^.

**Table 2:** Influence of PILs and cross-linker content in the chitosan gel beads on the physicochemical properties of the resulting chitosan aerogel particles. Notation: ρ_skel_, skeletal density (measured by helium pycnometry); ρ_env_, envelope density; ε, overall porosity. Values are expressed as mean followed by the standard deviation under parenthesis.

Entry	Particles	Diameter (mm)	ρ_skel_ (g/cm^3^)	ρ_env_ (g/cm^3^)	ε (%)	Overall Volume Shrinkage (%)
1	CHT	3.12 (0.1)	1.414 (0.030)	0.070 (0.015)	95.1 (1.0)	n.d.
2	CHT:P[DADMA]Cl_15%_	3.43 (0.1)	1.281 (0.044)	0.051 (0.010)	96.0 (0.8)	74.1 (5.4)
3	CHT:P[VBMPyr]Cl_15%_	3.44 (0.1)	1.254 (0.019)	0.052 (0.010)	95.9 (0.8)	66.3 (7.2)
4	CHT:P[VBA]Cl_15%_	3.41 (0.1)	1.304 (0.018)	0.057 (0.011)	95.6 (0.9)	68.7 (6.6)
5	CHT:P[DADMA][OAc]_15%_	3.27 (0.1)	1.299 (0.026)	0.070 (0.014)	94.6 (1.1)	69.7 (6.7)
6	CHT:P[DADMA]Cl_30%_	3.18 (0.1)	1.404 (0.058)	0.072 (0.015)	94.9 (1.1)	n.d.
7	CHT:P[VBMPyr]Cl_30%_	3.70 (0.1)	1.248 (0.026)	0.068 (0.012)	94.6 (0.9)	62.4 (7.5)
8	CHT:P[VBA]Cl_30%_	3.40 (0.1)	1.391 (0.018)	0.062 (0.012)	95.5 (0.9)	68.5 (6.7)
9	CHT:P[DADMA][OAc]_30%_	3.30 (0.1)	1.281 (0.043)	0.062 (0.012)	95.2 (1.0)	65.3 (7.7)
10	CHT:Glut_0.30%_	3.33 (0.1)	1.259 (0.017)	0.046 (0.010)	96.3 (0.8)	74.7 (5.4)
11	CHT:Glut_0.30%_:P[DADMA][OAc]_15%_	3.30 (0.1)	1.405 (0.015)	0.067 (0.013)	95.2 (0.9)	67.2 (7.2)
12	CHT:Glut_0.30%_:P[DADMA][OAc]_30%_	3.61 (0.1)	1.421 (0.010)	0.052 (0.010)	96.4 (0.7)	63.2 (7.5)
13	CHT:Glut_0.30%_:P[DADMA]Cl_15%_	3.43 (0.1)	1.390 (0.030)	0.055 (0.011)	96.0 (0.8)	55.1 (9.9)

nd: no data. Standard deviation was calculated using measurements of ca. 12 aerogel beads. See Table S1 in Supporting Information, for the complete set of samples.

**Table 3:** Textural properties evaluated by nitrogen adsorption-desorption tests of the chitosan aerogel particles. Notation: a_BET_, specific surface area by the BET method; V_P,BJH_, overall specific pore volume obtained by the BJH method; V_mes_, specific mesopore volume; V_MP_, specific macropore volume; D_P,BJH_, mean pore diameter by the BJH method.

**Entry**	**Particles**	**a_BET_ (m^2^/g)**	**V_P,BJH_ (cm^3^/g)**	**D_P,BJH_ (nm)**	**V_mes_** **(cm^3^/g)**	**V_MP_ (cm^3^/g)**
1	CHT	323	1.77	18.3	1.19	12.42
2	CHT:P[DADMA]Cl_15%_	332	1.51	15.1	1.05	17.72
3	CHT:P[VBMPyr]Cl_15%_	324	1.46	15.0	1.03	17.51
4	CHT:P[VBA]Cl_15%_	292	1.47	16.7	0.96	15.77
5	CHT:P[DADMA][OAc]_15%_	366	1.67	15.2	1.19	12.27
6	CHT:P[DADMA]Cl_30%_	449	2.23	16.3	1.40	11.85
7	CHT:P[VBMPyr]Cl_30%_	454	1.92	14.0	1.39	12.62
8	CHT:P[VBA]Cl_30%_	300	1.32	14.5	0.92	14.48
9	CHT:P[DADMA][OAc]_30%_	398	1.83	15.4	1.24	14.18
10	CHT:Glut_0.30%_	272	1.30	16.4	0.86	19.93
11	CHT:Glut_0.30%_:P[DADMA][OAc]_15%_	744	3.10	13.8	2.29	11.94
12	CHT:Glut_0.30%_:P[DADMA][OAc]_30%_	270	1.33	16.6	0.92	17.72
13	CHT:Glut_0.30%_:P[DADMA]Cl_15%_	344	1.47	14.2	1.02	16.40

See Table S2 in supporting information, for the complete set of samples.

**Table 4:** CO_2_ capture capacities of *AEROPILs* at 25 °C and 1 bar after exposure to CO_2_ for 10 min. Notation: n_CO2_, CO_2_ capture capacity.

Entry	Particles	n_CO2_ (mmol g^−1^)
1	CHT	0.57
2	CHT:P[DADMA]Cl_15%_	0.63
3	CHT:P[VBMPyr]Cl_15%_	0.60
4	CHT:P[VBA]Cl_15%_	0.53
5	CHT:P[DADMA][OAc]_15%_	0.60
6	CHT:P[DADMA]Cl_30%_	0.70
7	CHT:P[VBMPyr]Cl_30%_	0.59
8	CHT:P[VBA]Cl_30%_	0.64
9	CHT:P[DADMA][OAc]_30%_	0.62
10	CHT:Glut_0.30%_	0.67
11	CHT:Glut_0.30%_:P[DADMA][OAc]_15%_	0.57
12	CHT:Glut_0.30%_:P[DADMA][OAc]_30%_	0.64
13	CHT:Glut_0.30%_:P[DADMA]Cl_15%_	0.67

n_CO2_, CO_2_ capture capacity.

## Data Availability

Not applicable.

## Supplementary Materials

The following supporting information can be downloaded at: https://www.mdpi.com/article/10.3390/ijms23010200/s1.
