# Supplementary material for: The AEROPILs Generation: Novel Poly(Ionic Liquid)-Based Aerogels for CO2 Capture
Source: Int J Mol Sci. 2021 Dec 24;23(1):200. doi: 10.3390/ijms23010200 (PMC8745277; doi:10.3390/ijms23010200)
Supplement: Supplementary file 1 [file ijms-23-00200-s001.zip › ijms-1506214-supplementary.pdf]

Supporting information for

The *AEROPILs* Generation: Novel Poly(ionic liquid)-based Aerogels for CO_2 Capture

Raquel V. Barrulas[a], Clara López-Iglesias[b], Marcileia Zanatta[a,c], Teresa Casimiro[d], Gonzalo Mármol[e], Manuela Ribeiro Carrott[e], Carlos A. García-González[b], Marta C. Corvo[a*]

[a] i3N|*Cenimat, Department of Materials Science (DCM), NOVA School of Science and Technology, NOVA University Lisbon, 2829-516 Caparica, Portugal*

[b]*Department of Pharmacology, Pharmacy and Pharmaceutical Technology, I+D Farma Group (GI-1645), Faculty of Pharmacy and Health Research Institute of Santiago de Compostela (IDIS), Universidade de Santiago de Compostela, E-15782 Santiago de Compostela, Spain*

[c]*Present address: Institute of Advanced Materials (INAM), Universitat Jaume I, 12071 Castellón, Spain*

[d]*LAQV-REQUIMTE, Chemistry Department, NOVA School of Science and Technology, NOVA University Lisbon, 2829-516 Caparica, Portugal*

[e]*LAQV-REQUIMTE, Instituto de Investigação e Formação Avançada, Departamento de Química e Bioquímica, Escola de Ciências e Tecnologia, Colégio Luís António Verney, Universidade de Évora, 7000-671 Évora, Portugal*

*Corresponding author. Tel.: +351 21 294 8562; fax: +351 21 294 8558. *E-mail address*: marta.corvo@fct.unl.pt (Marta C. Corvo).

Table of contents

1 Synthesis of IL monomers – quaternization of 4-vinylbenzyl chloride 4

2 Synthesis of poly(ionic liquids) (PILs) 5

2.1 Ion exchange reaction – P[DADMA]Cl to P[DADMA][OAc] 5

3 General characterization of materials 6

3.1 Kinetics of the supercritical drying 8

3.2 Solid-state NMR analysis 8

3.3 Morphological and textural properties of the chitosan aerogels 9

3.4 BJH-pore size distribution and nitrogen adsorption-desorption isotherm 10

3.5 SEM imaging of the different *AEROPILs* 16

3.6 TGA curves 24

4 CO_2 capture 25

5 References 25

1 Synthesis of IL monomers – quaternization of 4-vinylbenzyl chloride

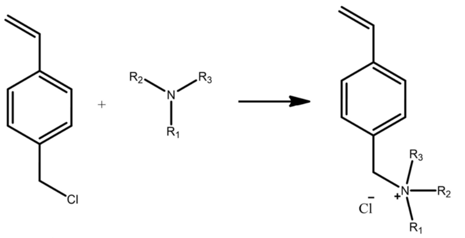

1 **2** **3**

	Conditions	Starting Materials
**2a**	Neat<br>50 °C	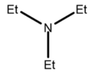
**2b**	MeCN<br>40 °C	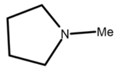

Figure S1 – Synthesis of vinylbenzyl chlorides 3.

Compound (1a) *p*-vinylbenzyltriethylammonium chloride ([VBA]Cl) and (1b) 1-methyl-1-(4'-vinylbenzyl)pyrrolidinium chloride ([VBMPyr]Cl) were synthesized according to procedures described in the literature.[1,2] [VBA]Cl was obtained from the reaction of 4-vinylbenzyl chloride (2.0 g, 13.1 mmol) and triethylamine (1.33 g, 13.1 mmol), at 50 ^{o}C under N_2 atmosphere and stirring for 19 h. The precipitated solid was collected and washed with diethyl ether. The final compound was obtained with a yield of 91%. [VBMPyr]Cl was obtained from the reaction of 4-vinylbenzyl chloride (1.00 g, 6.55 mmol), 1-methylpyrrolidine (0.56 g, 6.55 mmol) in MeCN (15 mL). The mixture was stirred at 40 ^{o}C under N_2 atmosphere and stirring for 22 h. The final product was collected and washed with ethyl acetate, being obtained in quantitative yield. The final structures of [VBA]Cl and [VBMPyr]Cl were confirmed by ^{1}H and ^{13}C NMR as well as by FTIR-ATR.

2 Synthesis of poly(ionic liquids) (PILs)

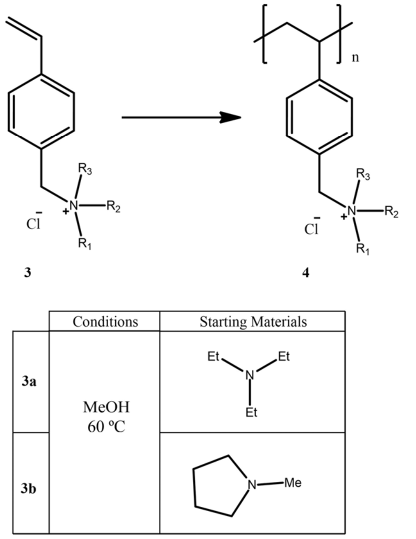

Figure S2 – Synthesis of PILs 4.

PILs from compound (1a) poly(*p*-vinylbenzyltriethylammonium) chloride (P[VBA]Cl) and (1b) poly(1-methyl-1-(4'-vinylbenzyl)pyrrolidinium) chloride (P[VBMPyr]Cl) were synthesized according to a procedure described in the literature.[3] P[VBA]Cl was obtained from the reaction of [VBA]Cl (1.02 g, 4 mmol), AIBN (22 mg, 0.13 mmol) in methanol (2.4 mL). The solution was then heated under N_2 atmosphere for 25 h at 85 ^oC. Polymerization was stopped by rapid cooling of the reaction mixture, and the polymer was precipitated with excess cold ethyl acetate, being the yield of 93%, with purity 93% (residual monomer determined by proton NMR). P[VBMPyr]Cl was obtained from the reaction of [VBMPyr]Cl (0.98 g, 4.13 mmol), AIBN (19.5 mg, 0.12 mmol) in methanol (2.5 mL). The solution was then heated under N_2 atmosphere for 22 h at 75 ^oC. Polymerization was stopped by rapid cooling of the reaction mixture, and the polymer was precipitated with excess cold ethyl acetate, being the yield 88%, with purity 100%.

2.1 Ion exchange reaction – P[DADMA]Cl to P[DADMA][OAc]

According to procedures described in the literature[4,5], poly(diallyldimethylammonium) acetate (P[DADMA][OAc]) was obtained through anion exchange reaction of the corresponding chloride salt as following: a solution (55 mL) of poly(diallyldimethylammonium) chloride (P[DADMA]Cl) (5.2 mmol) in water (0.1 mol L^{-1}) was eluted through a column containing 2.00 g of Amberlyst A-26 (OH^- form), previously prepared with 100 mL of sodium hydroxide solution (1 mol L^{-1}). The resulting solution (poly(diallyldimethylammonium) hydroxide) was collected into a 250 mL round-bottom flask and 1 equivalent (5.2 mmol) of acetic acid was added. The final

solution was stirred for 30 min and the solvent was removed under vacuum. P[DADMA][OAc] was obtained in a quantitative yield, after 24 h of drying at 60 ^{o}C under vacuum. The final structure of P[DADMA][OAc] was confirmed by ^{1}H and ^{13}C NMR as well as by FTIR-ATR.

3 General characterization of materials

NMR data were acquired at 298.2 K in a Bruker Avance III 400 spectrometer functioning at 400.15 MHz Larmor frequency for hydrogen and 100.61 MHz for carbon in a Bruker High Resolution BB-H&F-D-05DIFF probe. This spectrometer is equipped with a temperature control unit and a pulse field gradient unit capable of producing magnetic pulsed field gradients in the z-direction of 50.0 G/cm. Data were processed using Bruker Topspin 3.6.2 software. Spectra description is presented stating the deuterated solvent used and for each signal, chemical shift value, multiplicity, and integration. The following abbreviations were used to indicate signal multiplicity: s, singlet; d, doublet; t, triplet; q, quartet; m, multiplet. **^{1}H-NMR** experiments were acquired with 64 K time domain points over a spectral window of 8012.820 Hz (20.0244 ppm) centred at 2471.09 Hz (6.175 ppm) and with 32 scans per FID; the relaxation delay was set in 1.0 s. **^{13}C-NMR** experiments were acquired with 64 K time domain points over a spectral window of 24038.46 Hz (238.88 ppm) centred at 10060.84 (99.991 ppm) with variable scans per FID (1024 to 7168), the relaxation delay was variable, and from 1.5 s until 5.0 s.

ATR-FTIR spectra were recorded on a PerkinElmer Spectrum Two (Waltham, MA, USA) with 8 scans in the 4000 to 400 cm^{-1} range. The samples were placed in the sample holder directly in the IR laser beam. Spectra were processed using SpectraGryph 1.2.15 software. According to the most intense and characteristic vibrations data are shown as follows: maximum frequency absorption, v_{max}, in cm^{-1} (functional group), intensity and shape (s, strong; m, medium; w, weak; ar, aromatic).

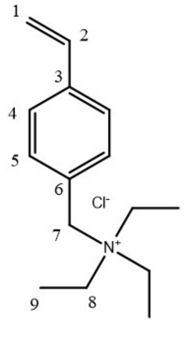

[VBA]Cl: **^{1}H-NMR** (400 MHz, DMSO-*d$_6$*) δ ppm 7.56 (m, 4H, $H_{4,4'}$+$H_{5,5'}$), 6.80 (dd, *J*=17.6 Hz and *J*=11.0 Hz, 1H, H_2), 5.96 (d, *J*=17.7 Hz, 1H, H_1), 5.38 (d, *J*=11.0 Hz, 1H, $H_{1'}$), 4.53 (s, 2H, H_7), 3.19 (q, *J*=7.2 Hz, 6H, $H_{8,8',8''}$), 1.31 (t, *J*=7.1 Hz, 9H, $H_{9,9',9''}$). **^{13}C-NMR** (101 MHz, DMSO-*d$_6$*) δ ppm 139.2 (C_3), 136.2 (C_2), 133.4 ($C_{5,5'}$), 127.8 (C_6), 127.0 ($C_{4,4'}$), 116.7 (C_1), 59.7 (C_7), 52.5 ($C_{8,8',8''}$), 8.1 ($C_{9,9',9''}$). **ATR-FTIR $v_{máx}$ cm^{-1}** 3401 (OH stretching, H_2O), 3075 (w), 2980 (s, -CH stretching), 1515 (m), 1478 (m, N-(CH_2-CH_3)$_3$), 1463 (s), 1411 (m, -C-N stretching), 1399 (s, C=C), 1295 (w, N-CH_2-), 1152 (m, ring -CH in-plane), 1010 (s), 945 (m), 862 (s, ar -CH), 808 (s, ar -CH).

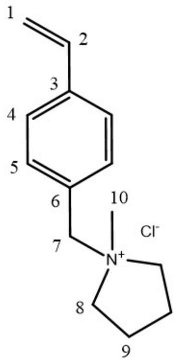

[VBMPyr]Cl: **^{1}H-NMR** (400 MHz, DMSO-*d_6*) δ ppm 7.59 (s, 4H, $H_{4,4'}$+$H_{5,5'}$), 6.80 (dd, *J*=17.7 Hz, *J*=10.9 Hz, 1H, H_2), 5.95 (dd, *J*=17.7 Hz, *J*=0.7 Hz, 1H, H_1), 5.37 (dd, *J*=10.9 Hz, *J*=0.7 Hz, 1H, $H_{1'}$), 4.68 (s, 2H, H_7), 3.63 (m, 2H, H_8), 3.43 (m, 2H, $H_{8'}$), 2.94 (s, 3H, H_{10}), 2.12 (m, 4H, $H_{9,9'}$). **^{13}C-NMR** (101 MHz, DMSO-*d_6*) δ ppm 139.1 (C_3), 136.3 (C_2), 133.3 ($C_{5,5'}$), 129.1 (C_6), 126.9 ($C_{4,4'}$), 116.5 (C_1), 65.0 (C_7), 62.9 ($C_{8,8'}$), 47.6 (C_{10}), 21.2 ($C_{9,9'}$). **ATR-FTIR $\nu_{máx}$ cm^{-1}** 3368 (br, s, OH stretching, H_2O), 2928 (s, -CH stretching), 2825 (m, -CH stretching), 1613 (m), 1513 (m), 1463 (s), 1427 (s, C=C), 1221 (m), 1028 (w), 932 (s), 862 (m, ar -CH), 833 (m, ar -CH), 634 (s), 555 (m).

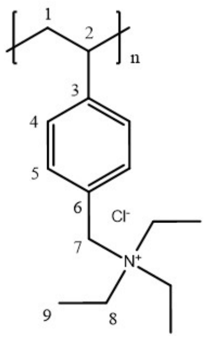

P[VBA]Cl: **^{1}H-NMR** (400 MHz, DMSO-*d_6*) δ ppm 7.58-7.26 (br, 2H, $H_{4,4'}$), 6.89-6.32 (br, 2H, $H_{5,5'}$), 5.05-4.33 (br, 2H, H_7), 3.34-3.13 (br, 6H, $H_{8,8',8''}$), 1.83-0.90 (m, 12H, H_1+H_2+$H_{9,9',9''}$). **^{13}C-NMR** (101 MHz, DMSO-*d_6*) δ ppm 136.2 (ar, C_5), 132.9 (br, C_4), 128.1 (br, $C_{5'}$), 126.5 (ar, $C_{4'}$), 60.2 (C_2+C_7), 52.4 ($C_{8,8',8''}$), 49.0 (C_1), 8.1 ($C_{9,9',9''}$). **ATR-FTIR $\nu_{máx}$ cm^{-1}** 3771 (br, m, OH stretching, H_2O), 2983 (m, -CH stretching), 2926 (m, -CH stretching), 1732 (m, ar), 1613 (w, ar), 1514 (w), 1484 (s), 1455 (s), 1397 (s), 1373 (s), 1243 (s), 1156 (m), 1097 (m), 1037 (s), 1011 (s), 847 (m, ar -CH), 824 (m, C-H bend benzene ring), 783 (s, C-H bend benzene ring), 588 (br, s).

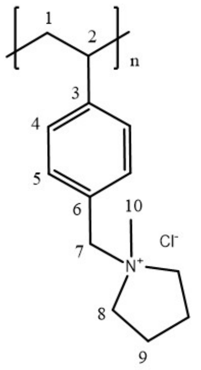

P[VBMPyr]Cl: **^{1}H-NMR** (400 MHz, DMSO-*d_6*) δ ppm 7.68-6.23 (br, 4H, $C_{4,4'}$+$C_{5,5'}$), 5.25-4.23 (br, 2H, C_7), 4.06-3.26 (br, 4H, $C_{8,8'}$), 3.22-2.75 (br, 3H, H_{10}), 2.34-2.04 (br, 4H, $H_{9,9'}$), 1.84-1.28 (br, 3H, H_1+H_2). **^{13}C-NMR** (101 MHz, DMSO-*d_6*) δ ppm 147.1 (br, C_3), 133.0 (br, $C_{5,5'}$), 128.1 (br, $C_{4,4'}$), 64.5 (C_7), 62.9 ($C_{8,8'}$), 49.0 (C_{10}), 47.5 (C_2), 24.9 (C_1), 21.2 ($C_{9,9'}$). **ATR-FTIR $\nu_{máx}$ cm^{-1}** 3369 (br, s, H_2O), 2924 (w, -CH stretching), 1613 (w, ar), 1513 (w, ar), 1463 (m), 1428 (w), 1012 (m), 934 (m), 860 (m, C-H bend benzene ring), 829 (s, C-H bend benzene ring), 553 (br, s).

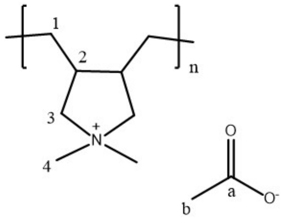

P[DADMA][OAc]: **^{1}H-NMR** (400 MHz, DMSO-*d_6*) δ ppm 3.34-3.02 (br, $H_{3,3'}$+$H_{4,4'}$), 2.20-2.00 (br, $H_{2,2'}$), 1.63 (s, 3H, H_b), 1.46-1.11 (br, $H_{1,1'}$). **^{13}C-NMR** (101 MHz, DMSO-*d_6*) δ ppm 175.3 (C_a), 69.8 ($C_{3,3'}$), 53.4 (C_4), 51.8 ($C_{4'}$), 38.3 (C_2), 37.6 ($C_{2'}$), 26.6 ($C_{1,1'}$), 25.6 (C_b). **ATR-FTIR $\nu_{máx}$ cm^{-1}** 3308.8 (s, -OH stretch), 1553.4 (s, O=C-O$^-$, acetate), 1475.3 (m, -CH in-plane bending).

3.1 Kinetics of the supercritical drying

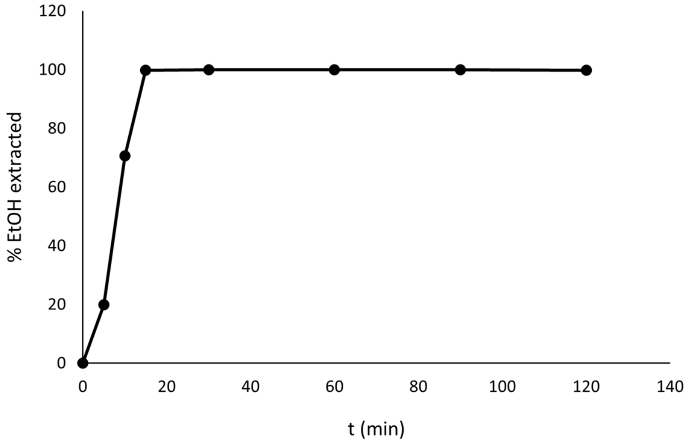

Figure S3 – Kinetics of the extraction of ethanol during the supercritical drying of *AEROPILs*.

3.2 Solid-state NMR analysis

Solid state ^{13}C MAS NMR spectra were acquired in a 11.7 T (500 MHz) AVANCE III Bruker spectrometer operating at 125 MHz (^{13}C), equipped with a BBO probe head. The samples were spun at the magic angle at a frequency of 5 kHz, using 4 mm diameter rotors at room temperature. The ^{13}C MAS NMR experiments were acquired with proton cross polarization and total suppression of sidebands (CP-TOSS) with a contact time of 2.0 ms, and a recycle delay of 5.0 s.

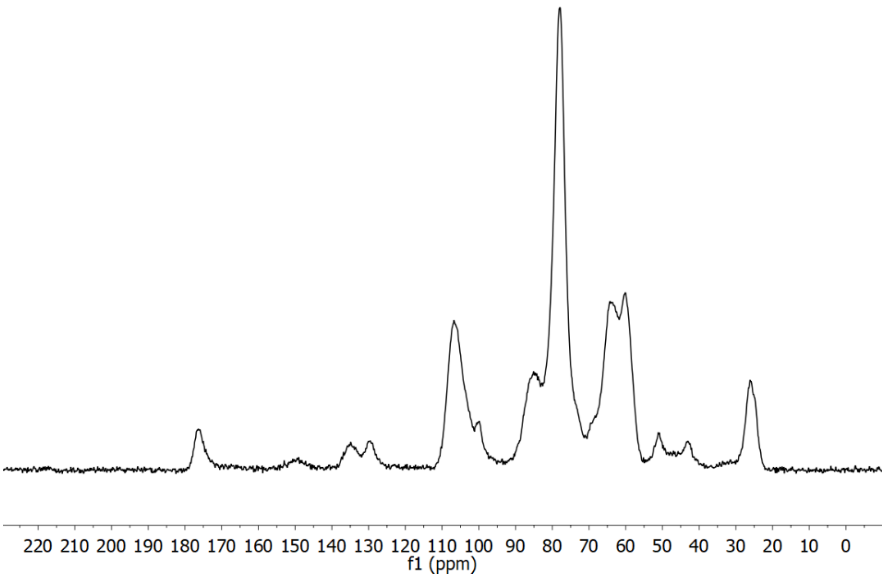

Figure S4 – ^{13}C CP-TOSS NMR spectra of CHT:Glut$_{4.0\%}$:P[VBMPyr]Cl$_{30\%}$ aerogel.

3.3 Morphological and textural properties of the chitosan aerogels

Table S1– *Influence of PILs and cross-linker of the chitosan gel beads on the physicochemical properties of the resulting chitosan aerogel particles. Notation: ρ_{skel}, skeletal density (measured by helium pycnometry); ρ_{env}, envelope density; ε, overall porosity. Values are expressed as mean followed by the standard deviation under parenthesis.*

Entry	Particles	Diameter (mm)	ρ_{skel} (g/cm^3)	ρ_{env} (g/cm^3)	ε (%)	Overall volume shrinkage (%)
1	CHT	3.12 (0.1)	1.414 (0.030)	0.070 (0.015)	95.1 (1.0)	n.d.
2	CHT:P[DADMA]$Cl_{15\%}$	3.43 (0.1)	1.281 (0.044)	0.051 (0.010)	96.0 (0.8)	74.1 (5.4)
3	CHT:P[VBMPyr]$Cl_{15\%}$	3.44 (0.1)	1.254 (0.019)	0.052 (0.010)	95.9 (0.8)	66.3 (7.2)
4	CHT:P[VBA]$Cl_{15\%}$	3.41 (0.1)	1.304 (0.018)	0.057 (0.011)	95.6 (0.9)	68.7 (6.6)
5	CHT:P[DADMA]$[OAc]_{15\%}$	3.27 (0.1)	1.299 (0.026)	0.070 (0.014)	94.6 (1.1)	69.7 (6.7)
6	CHT:P[DADMA]$Cl_{30\%}$	3.18 (0.1)	1.404 (0.058)	0.072 (0.015)	94.9 (1.1)	n.d.
7	CHT:P[VBMPyr]$Cl_{30\%}$	3.70 (0.1)	1.248 (0.026)	0.068 (0.012)	94.6 (0.9)	62.4 (7.5)
8	CHT:P[VBA]$Cl_{30\%}$	3.40 (0.1)	1.391 (0.018)	0.062 (0.012)	95.5 (0.9)	68.5 (6.7)
9	CHT:P[DADMA]$[OAc]_{30\%}$	3.30 (0.1)	1.281 (0.043)	0.062 (0.012)	95.2 (1.0)	65.3 (7.7)
10	CHT:$Glut_{0.13\%}$	3.09 (0.1)	1.426 (0.036)	0.054 (0.012)	96.2 (0.9)	67.4 (7.7)
11	CHT:$Glut_{0.20\%}$	3.40 (0.1)	1.437 (0.007)	0.043 (0.009)	97.0 (0.6)	61.4 (8.4)
12	CHT:$Glut_{0.30\%}$	3.33 (0.1)	1.259 (0.017)	0.046 (0.010)	96.3 (0.8)	74.7 (5.4)
13	CHT:$Glut_{0.13\%}$:P[DADMA]$[OAc]_{15\%}$	3.41 (0.1)	1.513 (0.010)	0.052 (0.010)	96.6 (0.7)	62.1 (8.2)
14	CHT:$Glut_{0.20\%}$:P[DADMA]$[OAc]_{15\%}$	3.13 (0.1)	1.482 (0.051)	0.063 (0.014)	95.8 (0.9)	65.5 (8.1)
15	CHT:$Glut_{0.30\%}$:P[DADMA]$[OAc]_{15\%}$	3.30 (0.1)	1.405 (0.015)	0.067 (0.013)	95.2 (0.9)	67.2 (7.2)
16	CHT:$Glut_{0.13\%}$:P[DADMA]$[OAc]_{30\%}$	3.30 (0.1)	1.395 (0.026)	0.060 (0.012)	95.7 (0.9)	64.0 (8.0)
17	CHT:$Glut_{0.20\%}$:P[DADMA]$[OAc]_{30\%}$	3.20 (0.1)	1.198 (0.015)	0.063 (0.013)	94.8 (1.1)	66.7 (7.6)
18	CHT:$Glut_{0.30\%}$:P[DADMA]$[OAc]_{30\%}$	3.61 (0.1)	1.421 (0.010)	0.052 (0.010)	96.4 (0.7)	63.2 (7.5)
19	CHT :$Glut_{0.13\%}$:P[DADMA]$Cl_{15\%}$	3.43 (0.1)	1.278 (0.041)	0.053 (0.010)	95.9 (0.8)	58.1 (9.2)
20	CHT :$Glut_{0.20\%}$:P[DADMA]$Cl_{15\%}$	3.43 (0.1)	1.409 (0.040)	0.058 (0.011)	95.9 (0.8)	58.1 (9.2)
21	CHT :$Glut_{0.30\%}$:P[DADMA]$Cl_{15\%}$	3.43 (0.1)	1.390 (0.030)	0.055 (0.011)	96.0 (0.8)	55.1 (9.9)

n.d.: no data. Standard deviation was calculated using measurements of *ca.* 12 aerogel beads.

Table S2– *Textural properties evaluated by nitrogen adsorption-desorption tests of the chitosan aerogel particles. Notation: a_{BET}, specific surface area by the BET method; $V_{P,BJH}$, overall specific pore volume obtained by the BJH-method; V_{mes}, specific mesopore volume; V_{MP}, specific macropore volume; $D_{P,BJH}$, mean pore diameter by the BJH-method.*

Entry	Particles	a_{BET} (m^2/g)	$V_{P,BJH}$ (cm^3/g)	$D_{P,BJH}$ (nm)	V_{mes} (cm^3/g)	V_{MP} (cm^3/g)
1	CHT	323	1.77	18.3	1.19	12.42
2	CHT:P[DADMA]$Cl_{15\%}$	332	1.51	15.1	1.05	17.72
3	CHT:P[VBMPyr]$Cl_{15\%}$	324	1.46	15.0	1.03	17.51
4	CHT:P[VBA]$Cl_{15\%}$	292	1.47	16.7	0.96	15.77
5	CHT:P[DADMA]$[OAc]_{15\%}$	366	1.67	15.2	1.19	12.27
6	CHT:P[DADMA]$Cl_{30\%}$	449	2.23	16.3	1.40	11.85
7	CHT:P[VBMPyr]$Cl_{30\%}$	454	1.92	14.0	1.39	12.62
8	CHT:P[VBA]$Cl_{30\%}$	300	1.32	14.5	0.92	14.48
9	CHT:P[DADMA]$[OAc]_{30\%}$	398	1.83	15.4	1.24	14.18
10	CHT:$Glut_{0.13\%}$	405	1.91	15.9	1.25	16.45
11	CHT:$Glut_{0.20\%}$	271	1.17	14.7	0.86	21.71
12	CHT:$Glut_{0.30\%}$	272	1.30	16.4	0.86	19.93
13	CHT:$Glut_{0.13\%}$:P[DADMA]$[OAc]_{15\%}$	369	1.56	14.1	1.15	17.57
14	CHT:$Glut_{0.20\%}$:P[DADMA]$[OAc]_{15\%}$	366	1.76	16.0	1.17	14.14
15	CHT:$Glut_{0.30\%}$:P[DADMA]$[OAc]_{15\%}$	744	3.10	13.8	2.29	11.94
16	CHT:$Glut_{0.13\%}$:P[DADMA]$[OAc]_{30\%}$	302	1.33	14.9	0.98	15.00
17	CHT:$Glut_{0.20\%}$:P[DADMA]$[OAc]_{30\%}$	331	1.45	15.0	1.04	14.12
18	CHT:$Glut_{0.30\%}$:P[DADMA]$[OAc]_{30\%}$	270	1.33	16.6	0.92	17.72
19	CHT :$Glut_{0.13\%}$:P[DADMA]$Cl_{15\%}$	575	2.48	14.5	1.73	16.49
20	CHT :$Glut_{0.20\%}$:P[DADMA]$Cl_{15\%}$	470	2.17	15.1	1.48	14.97
21	CHT :$Glut_{0.30\%}$:P[DADMA]$Cl_{15\%}$	344	1.47	14.2	1.02	16.40

3.4 BJH-pore size distribution and nitrogen adsorption-desorption isotherm

In Figures S5-S25 the lines in BJH-pore size distributions and nitrogen adsorption-desorption isotherms are only for visual guidance.

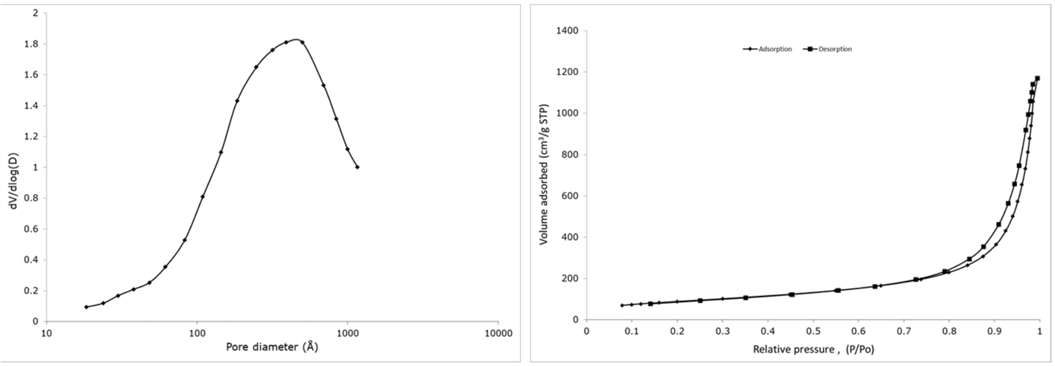

Figure S5 – BJH-pore size distribution and nitrogen adsorption-desorption isotherm for chitosan.

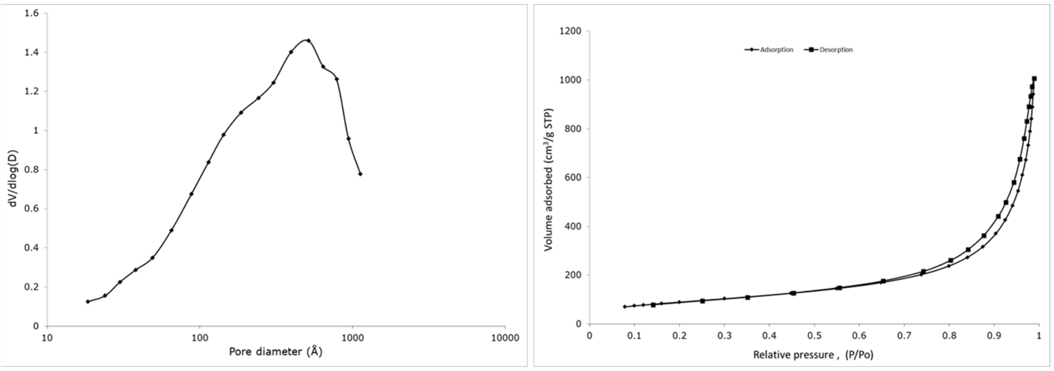

Figure S6 – BJH-pore size distribution and nitrogen adsorption-desorption isotherm for CHT:P[DADMA]Cl$_{15\%}$.

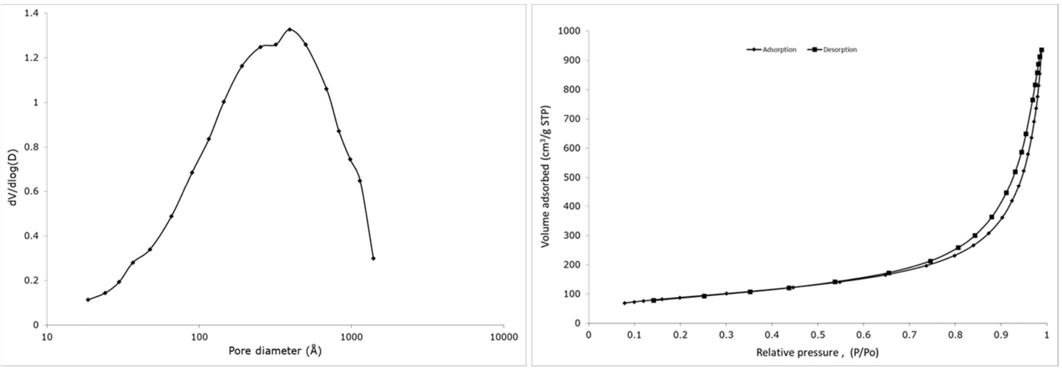

Figure S7 – BJH-pore size distribution and nitrogen adsorption-desorption isotherm for CHT:P[VBMPyr]Cl$_{15\%}$.

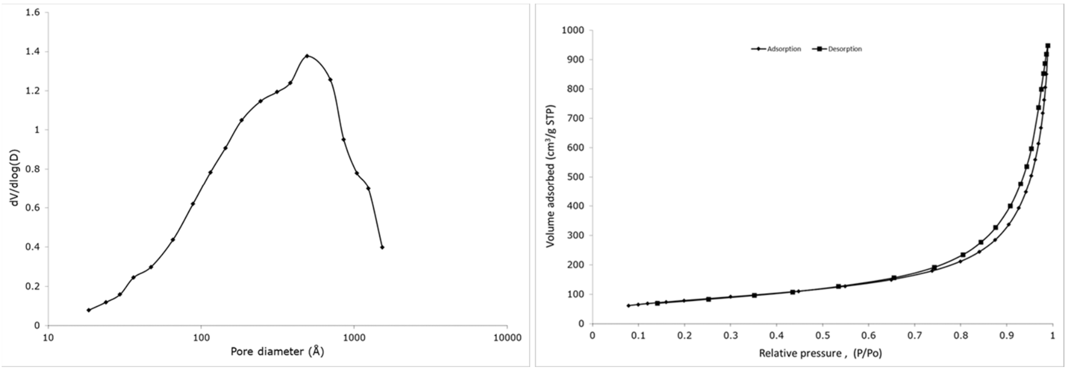

Figure S8 – BJH-pore size distribution and nitrogen adsorption-desorption isotherm for CHT:P[VBA]$Cl_{15\%}$.

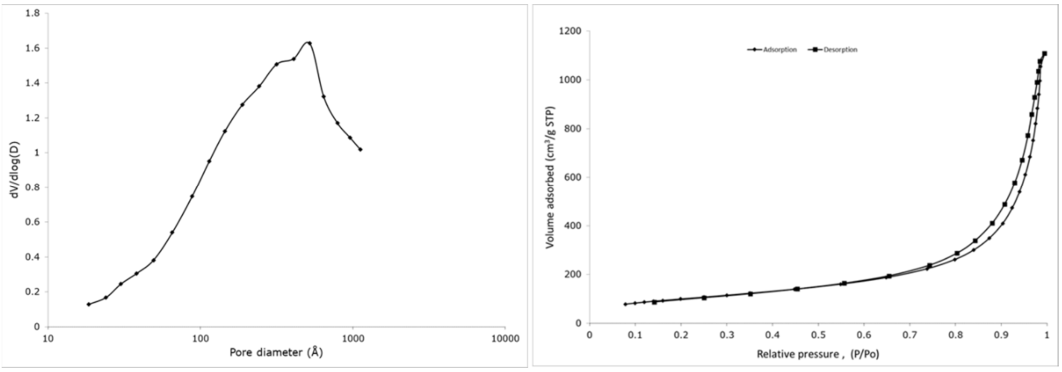

Figure S9 – BJH-pore size distribution and nitrogen adsorption-desorption isotherm for CHT:P[DADMA][OAc]$_{15\%}$.

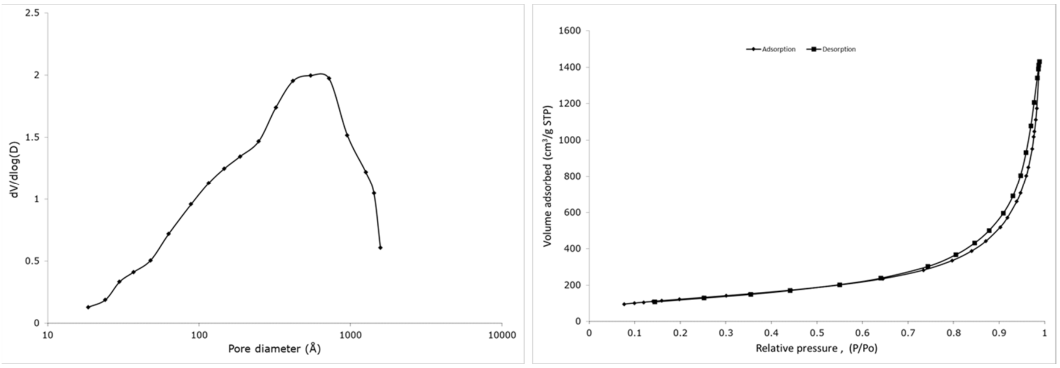

Figure S10 – BJH-pore size distribution and nitrogen adsorption-desorption isotherm for CHT:P[DADMA]$Cl_{30\%}$.

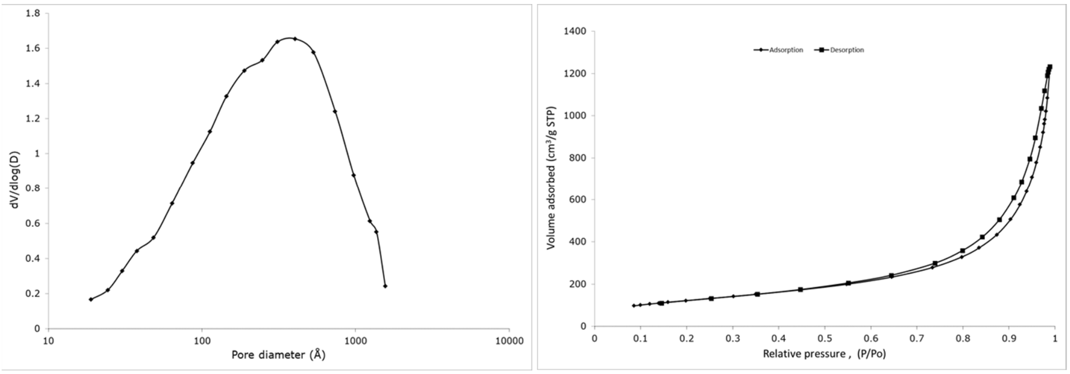

Figure S11 – BJH-pore size distribution and nitrogen adsorption-desorption isotherm for CHT:P[VBMPyr]$Cl_{30\%}$.

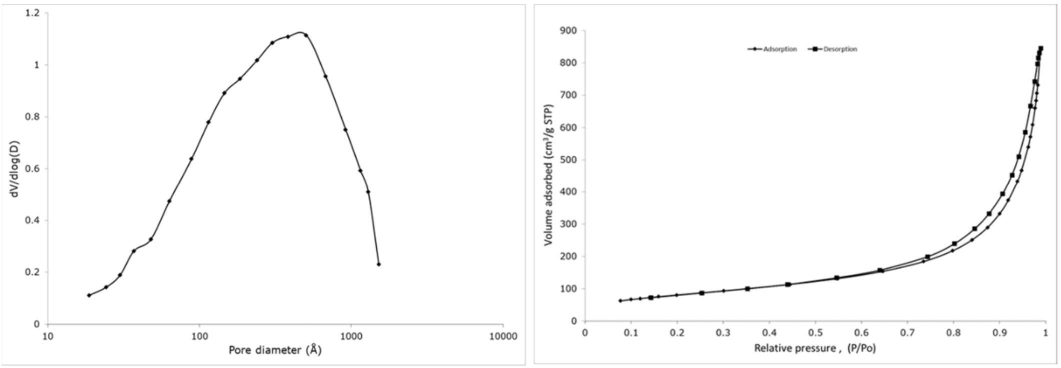

Figure S12 – BJH-pore size distribution and nitrogen adsorption-desorption isotherm for CHT:P[VBA]$Cl_{30\%}$.

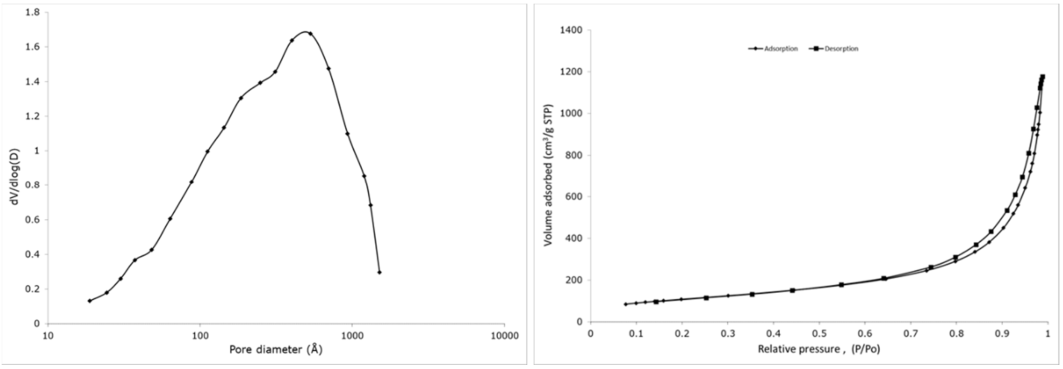

Figure S13 – BJH-pore size distribution and nitrogen adsorption-desorption isotherm for CHT:P[DADMA]$[OAc]_{30\%}$.

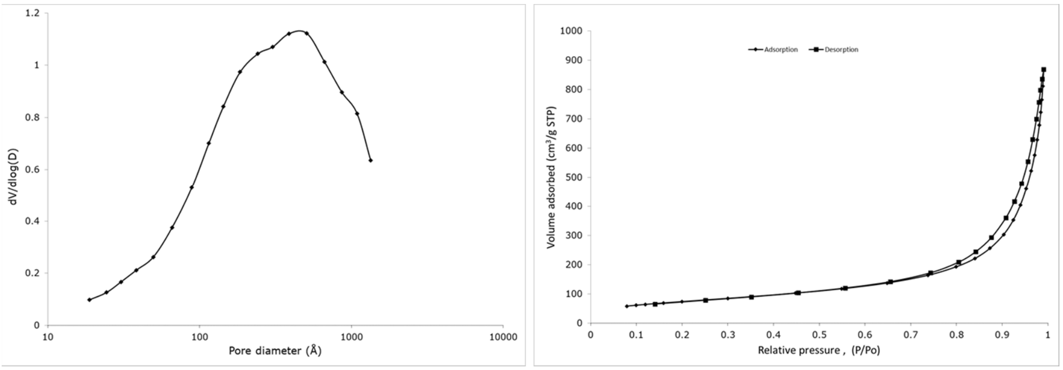

Figure S14 – BJH-pore size distribution and nitrogen adsorption-desorption isotherm for CHT:$Glut_{0.30\%}$.

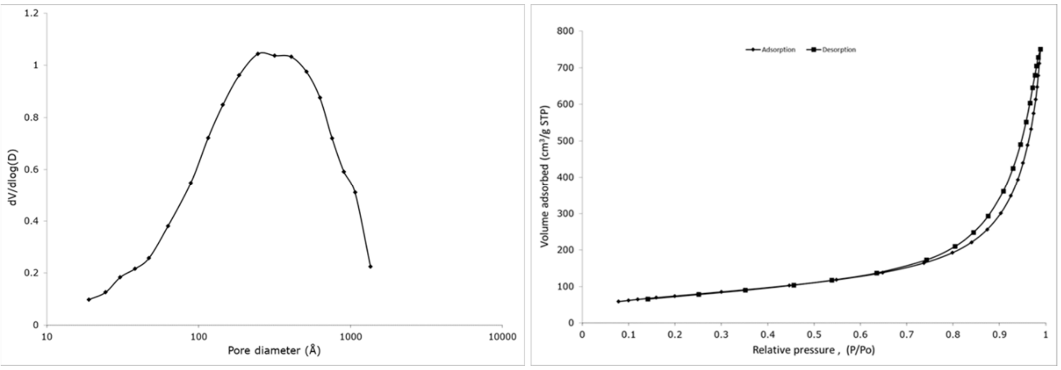

Figure S15 – BJH-pore size distribution and nitrogen adsorption-desorption isotherm for CHT:Glut$_{0.20\%}$.

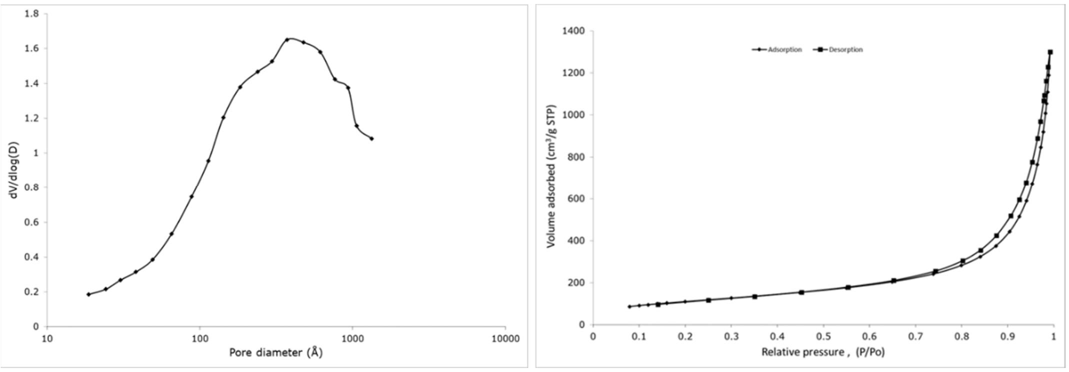

Figure S16 – BJH-pore size distribution and nitrogen adsorption-desorption isotherm for CHT:Glut$_{0.13\%}$.

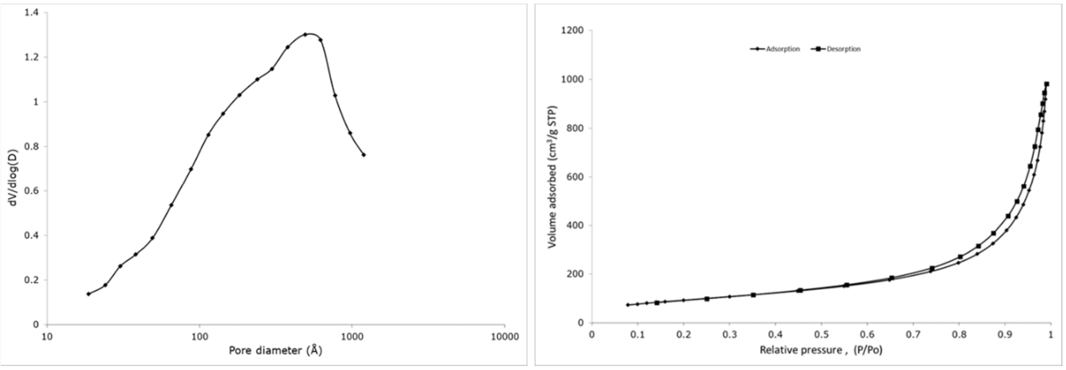

Figure S17 – BJH-pore size distribution and nitrogen adsorption-desorption isotherm for CHT:Glut$_{0.30\%}$:P[DADMA]Cl$_{15\%}$.

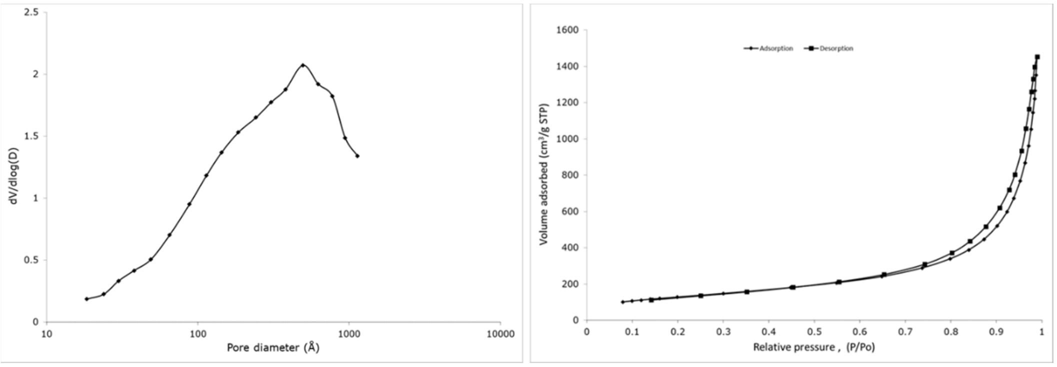

Figure S18 – BJH-pore size distribution and nitrogen adsorption-desorption isotherm for CHT:Glut$_{0.20\%}$:P[DADMA]Cl$_{15\%}$.

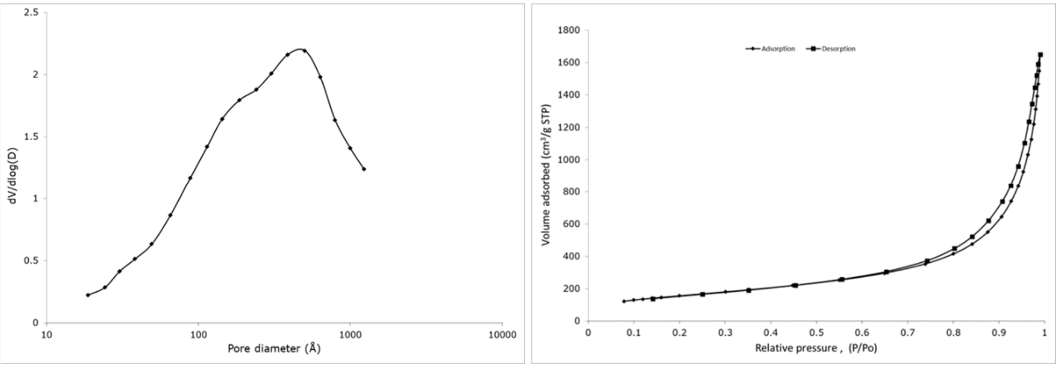

Figure S19 – BJH-pore size distribution and nitrogen adsorption-desorption isotherm for CHT:Glut$_{0.13\%}$:P[DADMA]Cl$_{15\%}$.

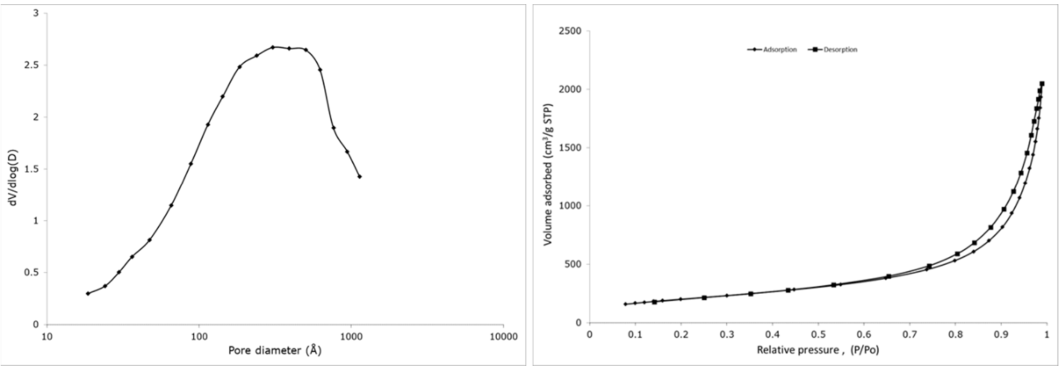

Figure S20 – BJH-pore size distribution and nitrogen adsorption-desorption isotherm for CHT:Glut$_{0.30\%}$:P[DADMA][OAc]$_{15\%}$.

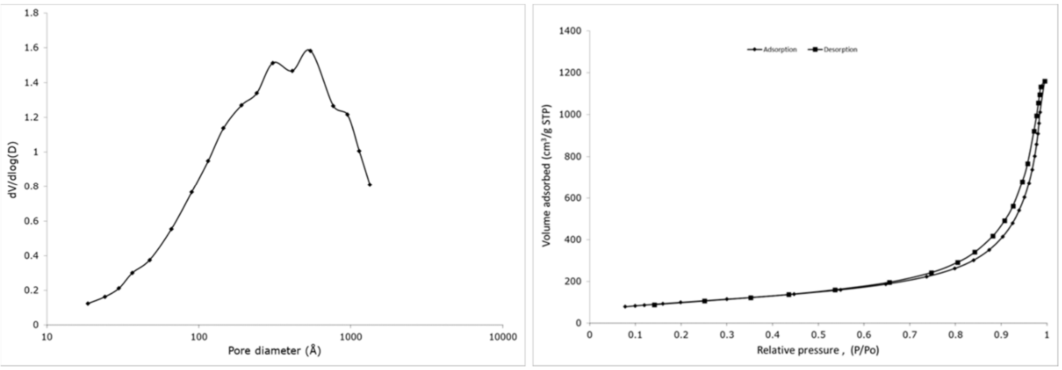

Figure S21 – BJH-pore size distribution and nitrogen adsorption-desorption isotherm for CHT:Glut$_{0.20\%}$:P[DADMA][OAc]$_{15\%}$.

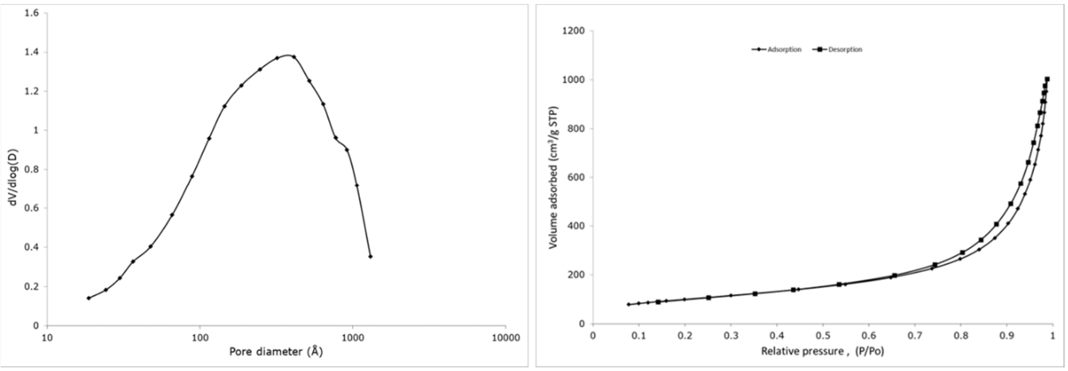

Figure S22 – BJH-pore size distribution and nitrogen adsorption-desorption isotherm for CHT:Glut$_{0.13\%}$:P[DADMA][OAc]$_{15\%}$.

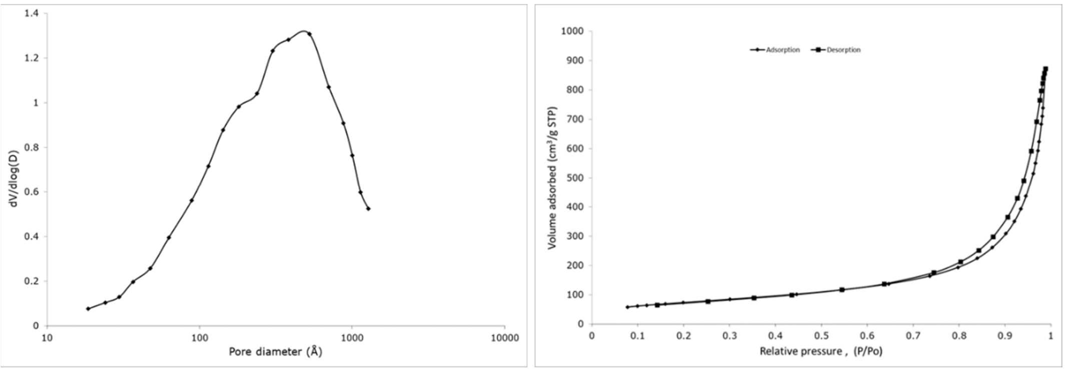

Figure S23 – BJH-pore size distribution and nitrogen adsorption-desorption isotherm for CHT:Glut$_{0.30\%}$:P[DADMA][OAc]$_{30\%}$.

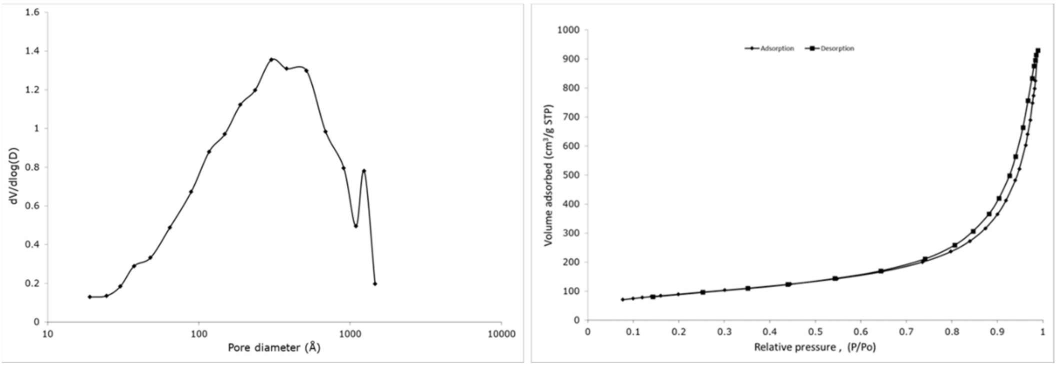

Figure S24 – BJH-pore size distribution and nitrogen adsorption-desorption isotherm for CHT:Glut$_{0.20\%}$:P[DADMA][OAc]$_{30\%}$.

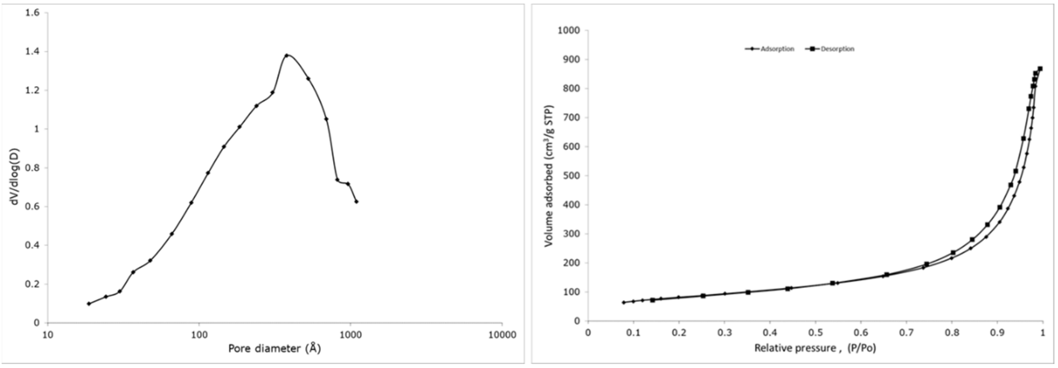

Figure S25 – BJH-pore size distribution and nitrogen adsorption-desorption isotherm for CHT:Glut$_{0.13\%}$:P[DADMA][OAc]$_{30\%}$.

3.5 SEM imaging of the different *AEROPILs*

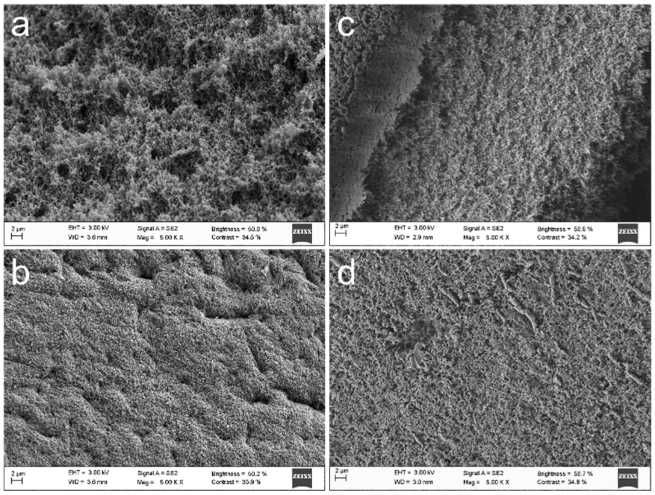

Figure S26 – Textural appearance of the (a,c) interior of beads CHT, CHT:P[VBA]Cl$_{15\%}$ respectively, and (b,d) surface of beads CHT, CHT:P[VBA]Cl$_{15\%}$ respectively, by SEM imaging (5.00 KX, 2 µm scale).

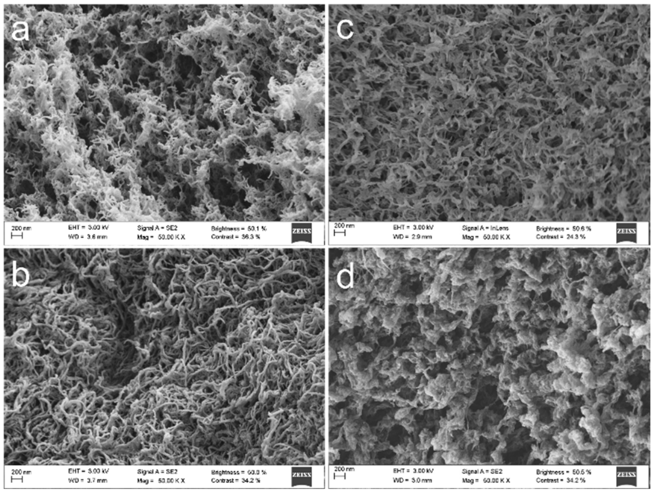

Figure S27 – Textural appearance of the (a,c) interior of beads CHT, CHT:P[VBA]Cl$_{15\%}$ respectively, and (b,d) surface of beads CHT, CHT:P[VBA]Cl$_{15\%}$ respectively, by SEM imaging (50.00 KX, 200 nm scale).

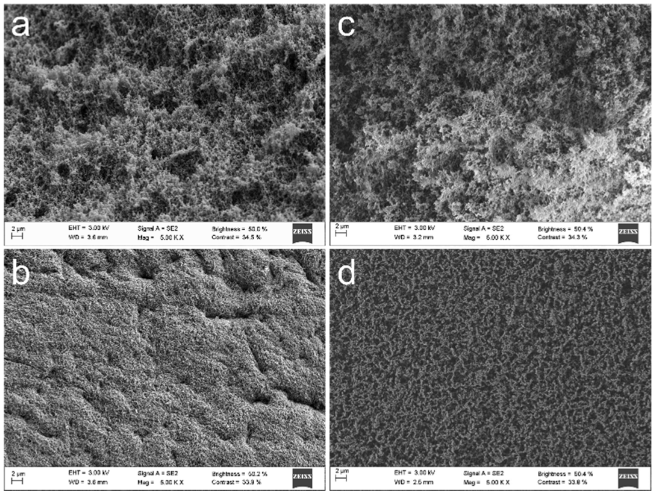

Figure S28 – Textural appearance of the (a,c) interior of beads CHT, CHT:P[VBMPyr]Cl$_{15\%}$ respectively, and (b,d) surface of beads CHT, CHT:P[VBMPyr]Cl$_{15\%}$ respectively, by SEM imaging (5.00 KX, 2 μm scale).

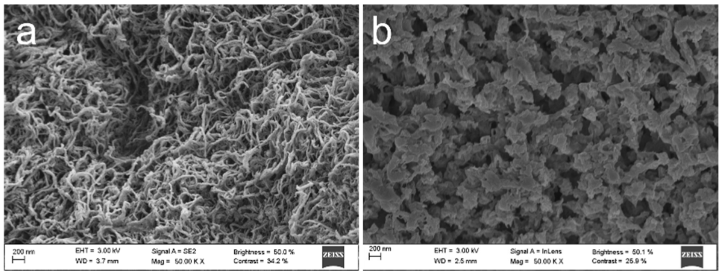

Figure S29 – Textural appearance of the (a,b) surface of beads CHT, CHT:P[VBMPyr]Cl$_{15\%}$ respectively, by SEM imaging (50.00 KX, 200 nm scale).

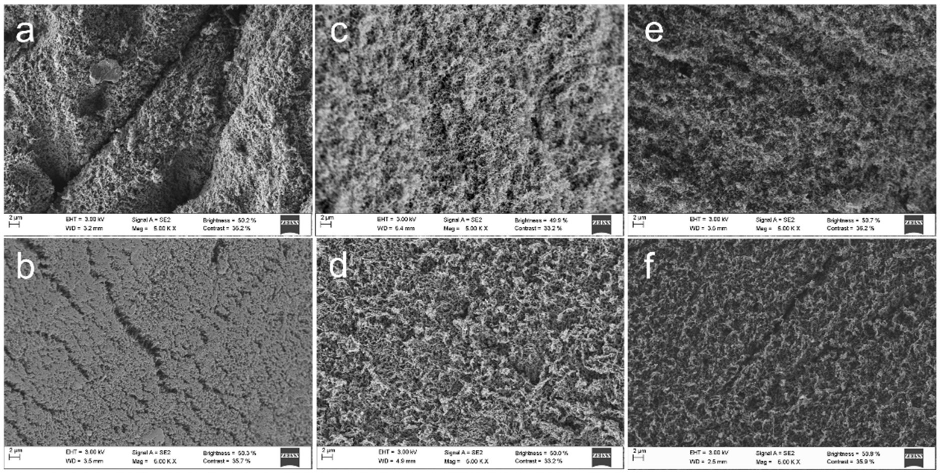

Figure S30 – Textural appearance of the (a,c,e) interior of beads CHT:Glut$_{0.13\%}$, CHT:Glut$_{0.13\%}$:P[DADMA][OAc]$_{15\%}$, CHT: P[DADMA][OAc]$_{15\%}$ respectively, and (b,d,f) surface of beads CHT:Glut$_{0.13\%}$, CHT:Glut$_{0.13\%}$:P[DADMA][OAc]$_{15\%}$, CHT: P[DADMA][OAc]$_{15\%}$ respectively, by SEM imaging (5.00 KX, 2 µm scale).

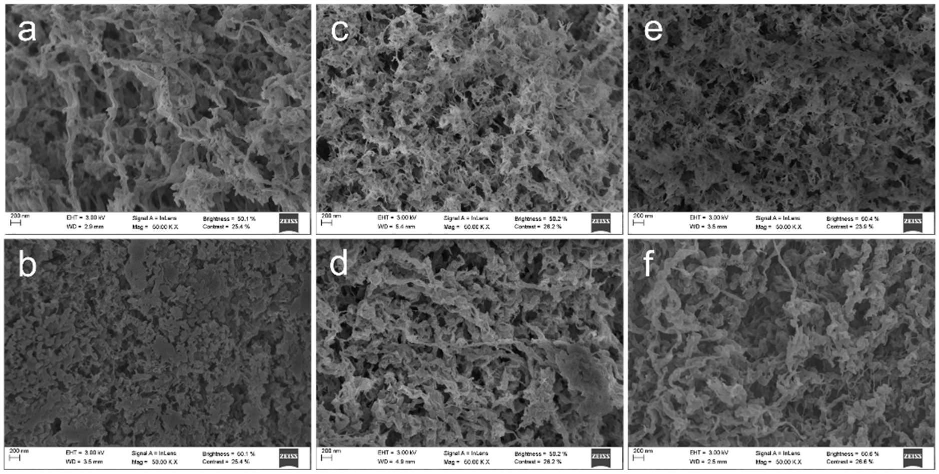

Figure S31 – Textural appearance of the (a,c,e) interior of beads CHT:Glut$_{0.13\%}$, CHT:Glut$_{0.13\%}$:P[DADMA][OAc]$_{15\%}$, CHT: P[DADMA][OAc]$_{15\%}$ respectively, and (b,d,f) surface of beads CHT:Glut$_{0.13\%}$, CHT:Glut$_{0.13\%}$:P[DADMA][OAc]$_{15\%}$, CHT: P[DADMA][OAc]$_{15\%}$ respectively, by SEM imaging (50.00 KX, 200 nm scale).

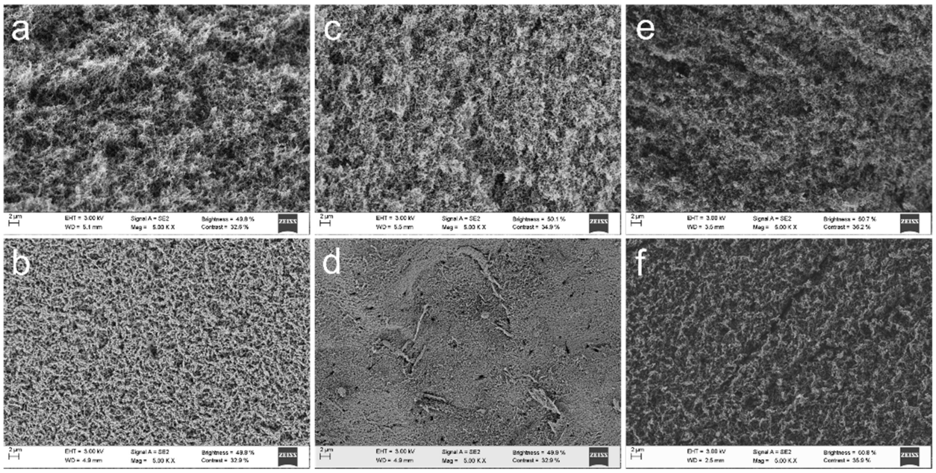

Figure S32 – Textural appearance of the (a,c,e) interior of beads CHT:Glut$_{0.20\%}$, CHT:Glut$_{0.20\%}$:P[DADMA][OAc]$_{15\%}$, CHT: P[DADMA][OAc]$_{15\%}$ respectively, and (b,d,f) surface of beads CHT:Glut$_{0.20\%}$, CHT:Glut$_{0.20\%}$:P[DADMA][OAc]$_{15\%}$, CHT: P[DADMA][OAc]$_{15\%}$ respectively, by SEM imaging (5.00 KX, 2 µm scale).

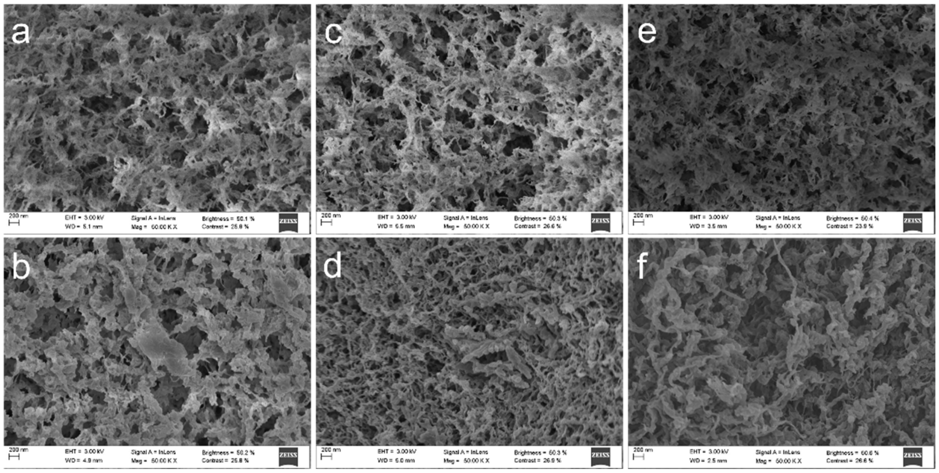

Figure S33 – Textural appearance of the (a,c,e) interior of beads CHT:Glut$_{0.20\%}$, CHT:Glut$_{0.20\%}$:P[DADMA][OAc]$_{15\%}$, CHT: P[DADMA][OAc]$_{15\%}$ respectively, and (b,d,f) surface of beads CHT:Glut$_{0.20\%}$, CHT:Glut$_{0.20\%}$:P[DADMA][OAc]$_{15\%}$, CHT: P[DADMA][OAc]$_{15\%}$ respectively, by SEM imaging (50.00 KX, 200 nm scale).

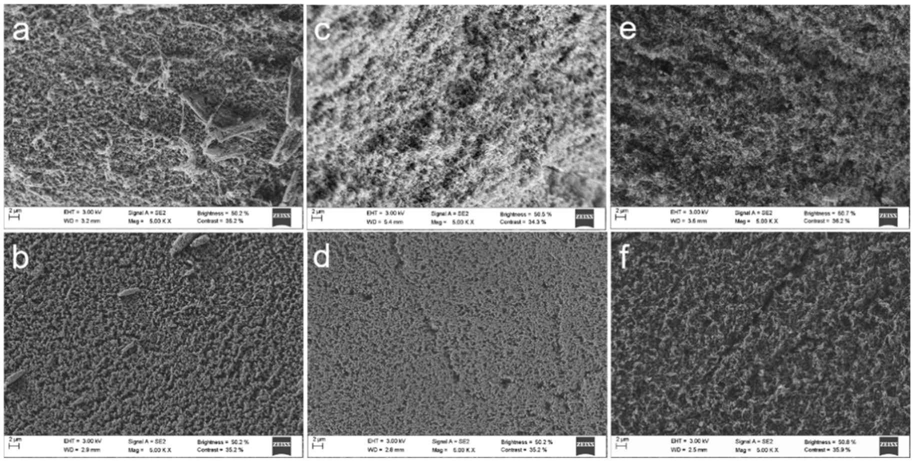

Figure S34 – Textural appearance of the (a,c,e) interior of beads CHT:Glut$_{0.30\%}$, CHT:Glut$_{0.30\%}$:P[DADMA][OAc]$_{15\%}$, CHT: P[DADMA][OAc]$_{15\%}$ respectively, and (b,d,f) surface of beads CHT:Glut$_{0.30\%}$, CHT:Glut$_{0.30\%}$:P[DADMA][OAc]$_{15\%}$, CHT: P[DADMA][OAc]$_{15\%}$ respectively, by SEM imaging (5.00 KX, 2 µm scale).

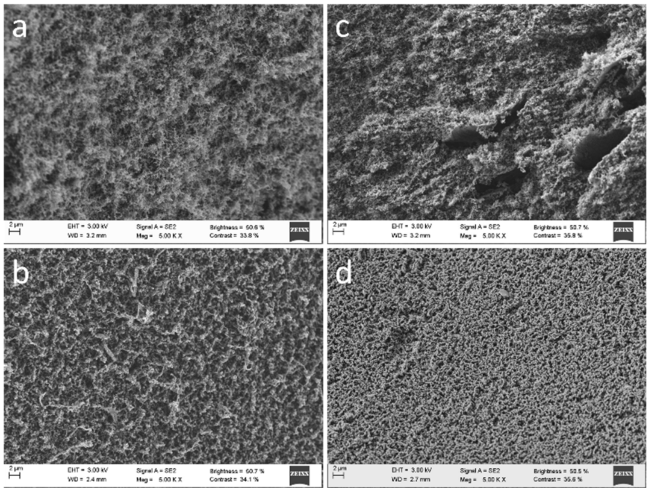

Figure S35 – Textural appearance of the (a,c) interior of beads CHT:P[DADMA]Cl$_{15\%}$, CHT:P[DADMA]Cl$_{30\%}$ respectively, and (b,d) surface of beads CHT:P[DADMA]Cl$_{15\%}$, CHT:P[DADMA]Cl$_{30\%}$ respectively, by SEM imaging (5.00 KX, 2 µm scale).

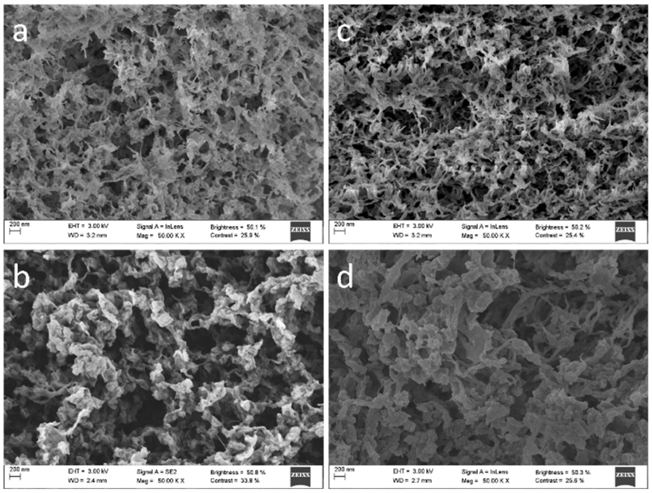

Figure S36 – Textural appearance of the (a,c) interior of beads CHT:P[DADMA]$Cl_{15\%}$, CHT:P[DADMA]$Cl_{30\%}$ respectively, and (b,d) surface of beads CHT:P[DADMA]$Cl_{15\%}$, CHT:P[DADMA]$Cl_{30\%}$ respectively, by SEM imaging (50.00 KX, 200 nm scale).

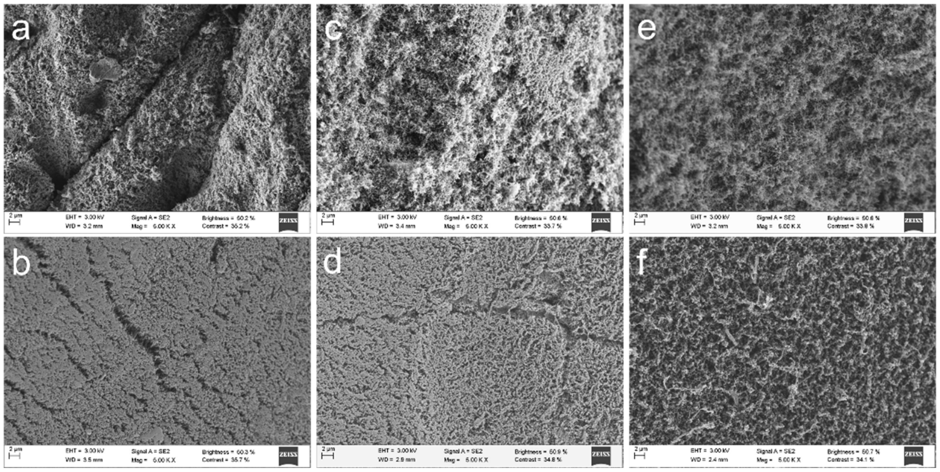

Figure S37 – Textural appearance of the (a,c,e) interior of beads CHT:$Glut_{0.13\%}$, CHT:$Glut_{0.13\%}$:P[DADMA]$Cl_{15\%}$, CHT: P[DADMA]$Cl_{15\%}$ respectively, and (b,d,f) surface of beads CHT:$Glut_{0.13\%}$, CHT:$Glut_{0.13\%}$:P[DADMA]$Cl_{15\%}$, CHT: P[DADMA]$Cl_{15\%}$ respectively, by SEM imaging (5.00 KX, 2 μm scale).

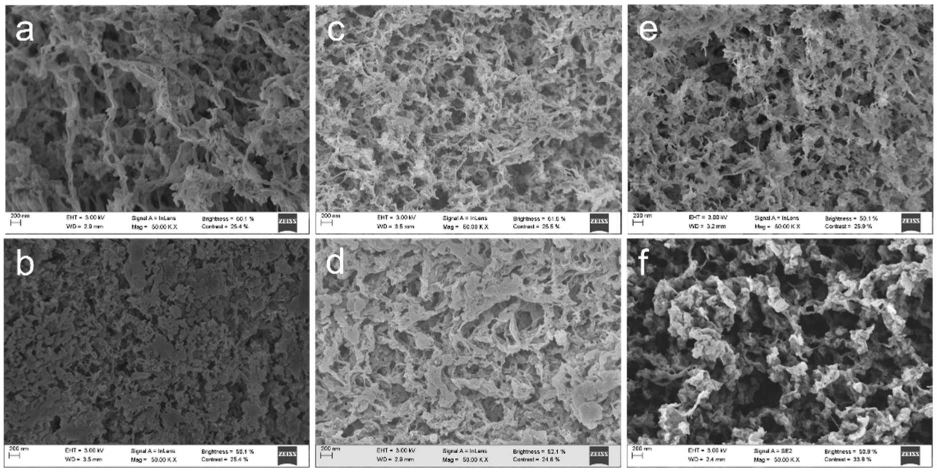

Figure S38 – Textural appearance of the (a,c,e) interior of beads CHT:Glut$_{0.13\%}$, CHT:Glut$_{0.13\%}$:P[DADMA]Cl$_{15\%}$, CHT: P[DADMA]Cl$_{15\%}$ respectively, and (b,d,f) surface of beads CHT:Glut$_{0.13\%}$, CHT:Glut$_{0.13\%}$:P[DADMA]Cl$_{15\%}$, CHT: P[DADMA]Cl$_{15\%}$ respectively, by SEM imaging (50.00 KX, 200 nm scale).

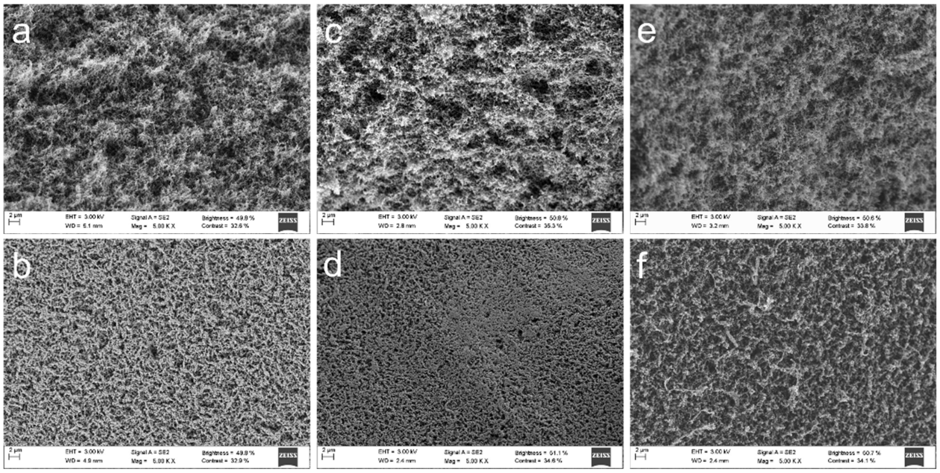

Figure S39 – Textural appearance of the (a,c,e) interior of beads CHT:Glut$_{0.20\%}$, CHT:Glut$_{0.20\%}$:P[DADMA]Cl$_{15\%}$, CHT: P[DADMA]Cl$_{15\%}$ respectively, and (b,d,f) surface of beads CHT:Glut$_{0.20\%}$, CHT:Glut$_{0.20\%}$:P[DADMA]Cl$_{15\%}$, CHT: P[DADMA]Cl$_{15\%}$ respectively, by SEM imaging (5.00 KX, 2 µm scale).

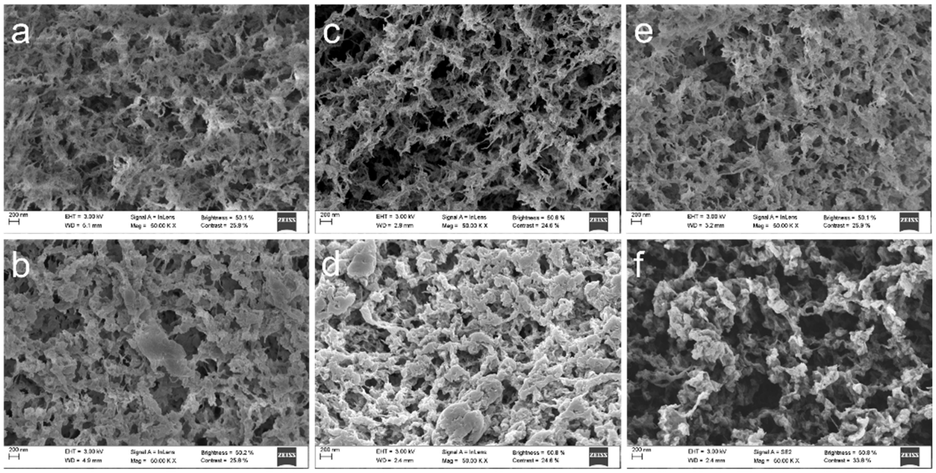

Figure S40 – Textural appearance of the (a,c,e) interior of beads CHT:Glut$_{0.20\%}$, CHT:Glut$_{0.20\%}$:P[DADMA]Cl$_{15\%}$, CHT: P[DADMA]Cl$_{15\%}$ respectively, and (b,d,f) surface of beads CHT:Glut$_{0.20\%}$, CHT:Glut$_{0.20\%}$:P[DADMA]Cl$_{15\%}$, CHT: P[DADMA]Cl$_{15\%}$ respectively, by SEM imaging (50.00 KX, 200 nm scale).

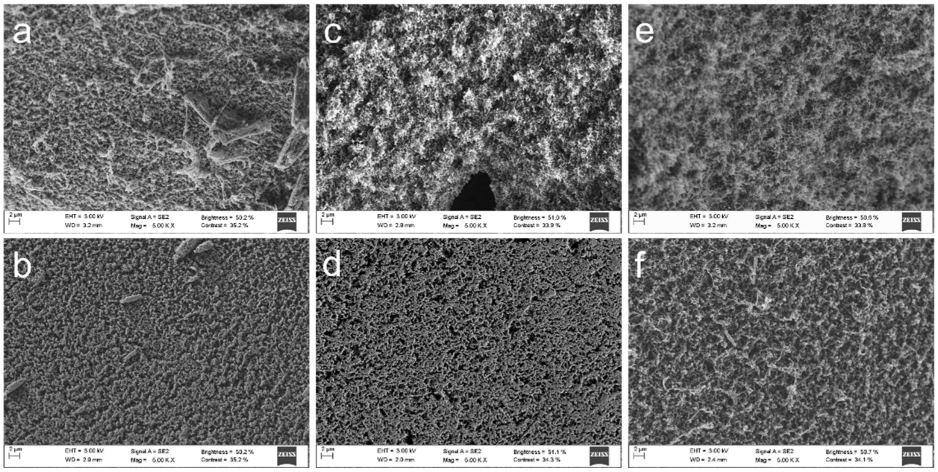

Figure S41 – Textural appearance of the (a,c,e) interior of beads CHT:Glut$_{0.30\%}$, CHT:Glut$_{0.30\%}$:P[DADMA]Cl$_{15\%}$, CHT: P[DADMA]Cl$_{15\%}$ respectively, and (b,d,f) surface of beads CHT:Glut$_{0.30\%}$, CHT:Glut$_{0.30\%}$:P[DADMA]Cl$_{15\%}$, CHT: P[DADMA]Cl$_{15\%}$ respectively, by SEM imaging (5.00 KX, 2 µm scale).

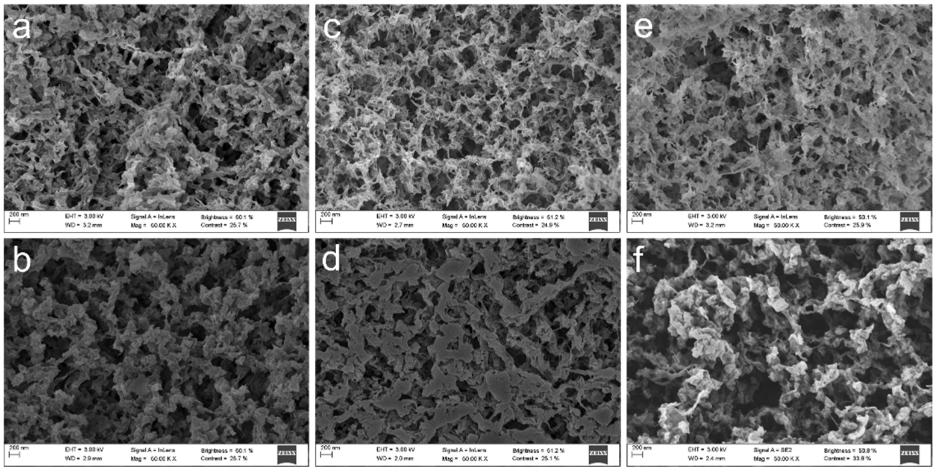

Figure S42 – Textural appearance of the (a,c,e) interior of beads CHT:Glut$_{0.30\%}$, CHT:Glut$_{0.30\%}$:P[DADMA]Cl$_{15\%}$, CHT: P[DADMA]Cl$_{15\%}$ respectively, and (b,d,f) surface of beads CHT:Glut$_{0.30\%}$, CHT:Glut$_{0.30\%}$:P[DADMA]Cl$_{15\%}$, CHT: P[DADMA]Cl$_{15\%}$ respectively, by SEM imaging (50.00 KX, 200 nm scale).

3.6 TGA curves

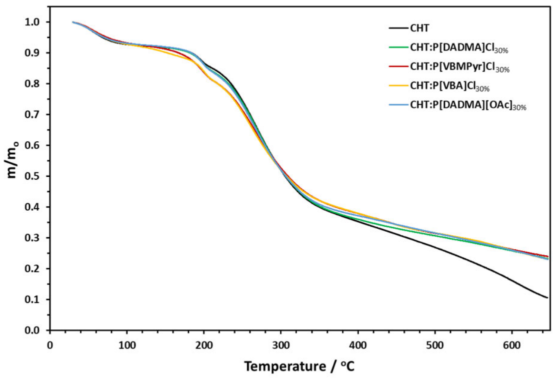

Figure S43– Representative TGA curves of CHT aerogel beads and *AEROPILs* (m_o is the mass at 30 °C).

4 CO_2 capture

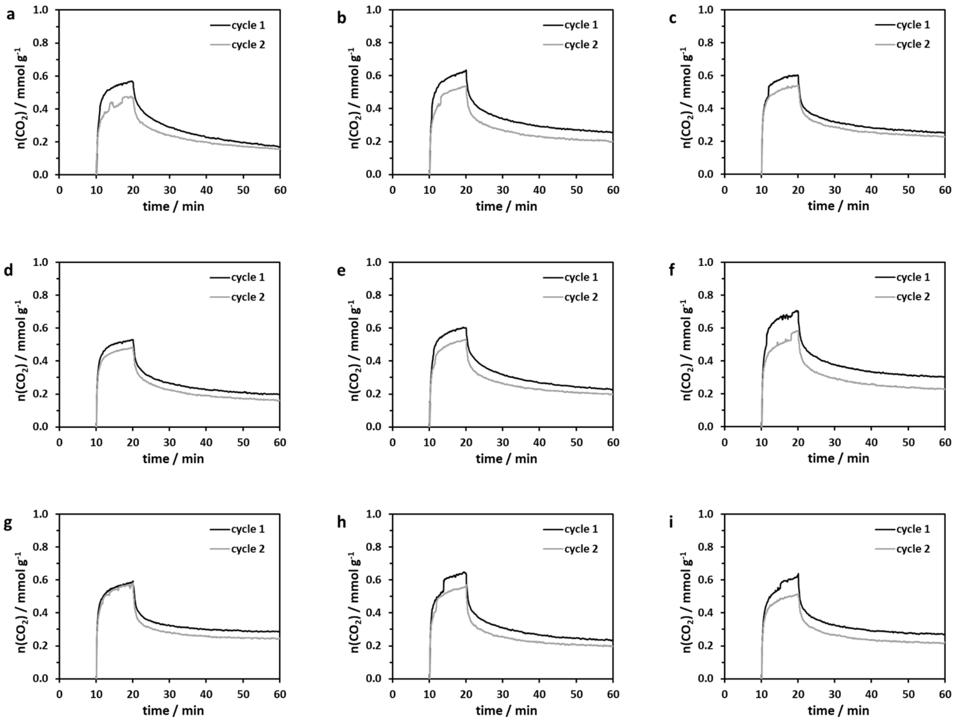

Figure S44 – Adsorption-desorption of CO_2, at 25 °C and 1 bar, on (a) CHT aerogel beads, (b) CHT:P[DADMA]$Cl_{15\%}$, (c) CHT:P[VBMPyr]$Cl_{15\%}$, (d) CHT:P[VBA]$Cl_{15\%}$, (e) CHT:P[DADMA][OAc]$_{15\%}$, (f) CHT:P[DADMA]$Cl_{30\%}$, (g) CHT:P[VBMPyr]$Cl_{30\%}$, (h) CHT:P[VBA]$Cl_{30\%}$ and (i) CHT:P[DADMA][OAc]$_{30\%}$.

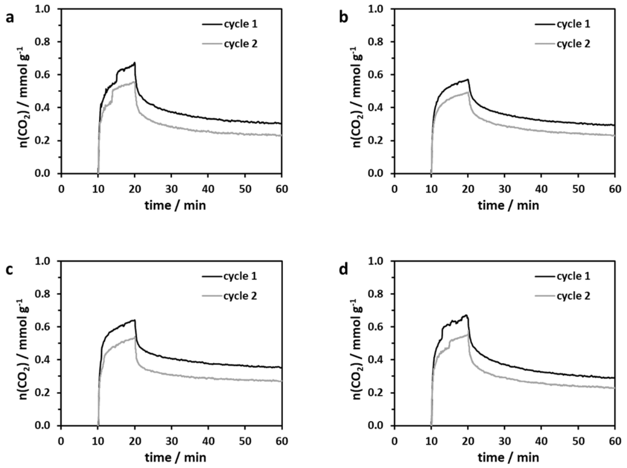

Figure S45 – Adsorption-desorption of CO_2, at 25 °C and 1 bar, on (a) CHT:Glut$_{0.30\%}$, (b) CHT:Glut$_{0.30\%}$:P[DADMA][OAc]$_{15\%}$, (c) CHT:Glut$_{0.30\%}$:P[DADMA][OAc]$_{30\%}$ and (d) CHT :Glut$_{0.30\%}$:P[DADMA]$Cl_{15\%}$.

5 References

[1] J. Tang, H. Tang, W. Sun, M. Radosz, Y. Shen, Low-pressure CO2 sorption in ammonium-

based poly(ionic liquid)s, Polymer (Guildf). 46 (2005) 12460–12467. doi:10.1016/j.polymer.2005.10.082.

[2] A. Zajac, A. Szpecht, D. Zielinski, K. Rola, J. Hoppe, K. Komorowska, M. Smiglak, Synthesis and characterization of potentially polymerizable amine-derived ionic liquids bearing 4-vinylbenzyl group, J. Mol. Liq. 283 (2019) 427–439. doi:10.1016/j.molliq.2019.03.061.

[3] N.K. Mogha, N. Yadav, A. Sindhu, P. Venkatesu, Does poly(ionic liquid) modulate the non-covalent interactions of chicken egg white lysozyme? Elucidation of biomolecular interactions between biomolecules and macromolecular solvents, New J. Chem. 43 (2019) 16759–16766. doi:10.1039/c9nj04078a.

[4] M. Zanatta, M. Lopes, E.J. Cabrita, C.E.S. Bernardes, M.C. Corvo, Handling CO2sorption mechanism in PIL@IL composites, J. CO2 Util. 41 (2020) 101225. doi:10.1016/j.jcou.2020.101225.

[5] M. Zanatta, A.L. Girard, G. Marin, G. Ebeling, F.P. Dos Santos, C. Valsecchi, H. Stassen, P.R. Livotto, W. Lewis, J. Dupont, Confined water in imidazolium based ionic liquids: A supramolecular guest@host complex case, Phys. Chem. Chem. Phys. 18 (2016) 18297–18304. doi:10.1039/c6cp03112a.
